# Supplementary material for: Metabolism-Related Gene Pairs to Predict the Clinical Outcome and Molecular Characteristics of Early Hepatocellular Carcinoma
Source: Cancers (Basel). 2022 Aug 16;14(16):3957. doi: 10.3390/cancers14163957 (PMC9406433; doi:10.3390/cancers14163957)
Supplement: Supplementary file 1 [file cancers-14-03957-s001.zip › cancers-1853279-supplementary.pdf]

cancers

MDPI

Supplementary Material

Metabolism-Related Gene Pairs to Predict the Clinical Outcome and Molecular Characteristics of Early Hepatocellular Carcinoma

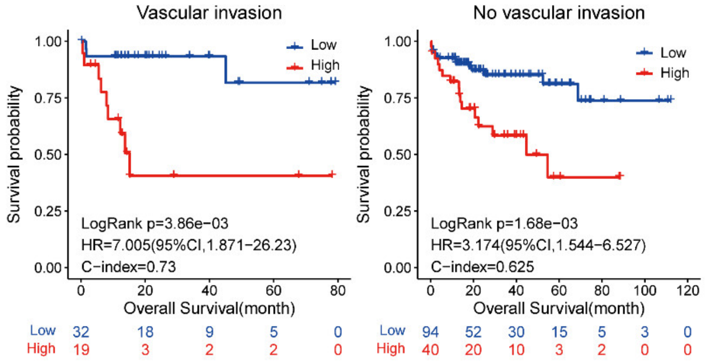

Chi-Squared test
(X^2=0.62, adjust *p*=0.43)

	High-risk	Low-risk
Vascular invasion	19	32
No vascular invasion	40	94

Figure S1. Analysis of 10-GPS in samples with and without vascular invasion from HCC197. OS of two prognostic groups in samples with or without vascular invasion. Statistical significance *p*-values were calculated by Chi-Squared test.

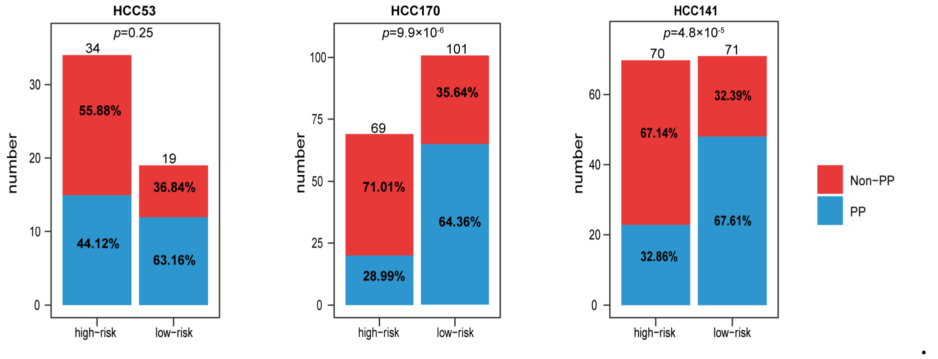

Figure S2. The distribution of PP and non-PP subtypes along with two prognostic groups. Patients were classified into the PP and non-PP subtypes based on the median value of the principal component analysis (PCA) of eight representative proliferation genes. Statistical significance *p*-values were calculated by Fisher′ s exact test.

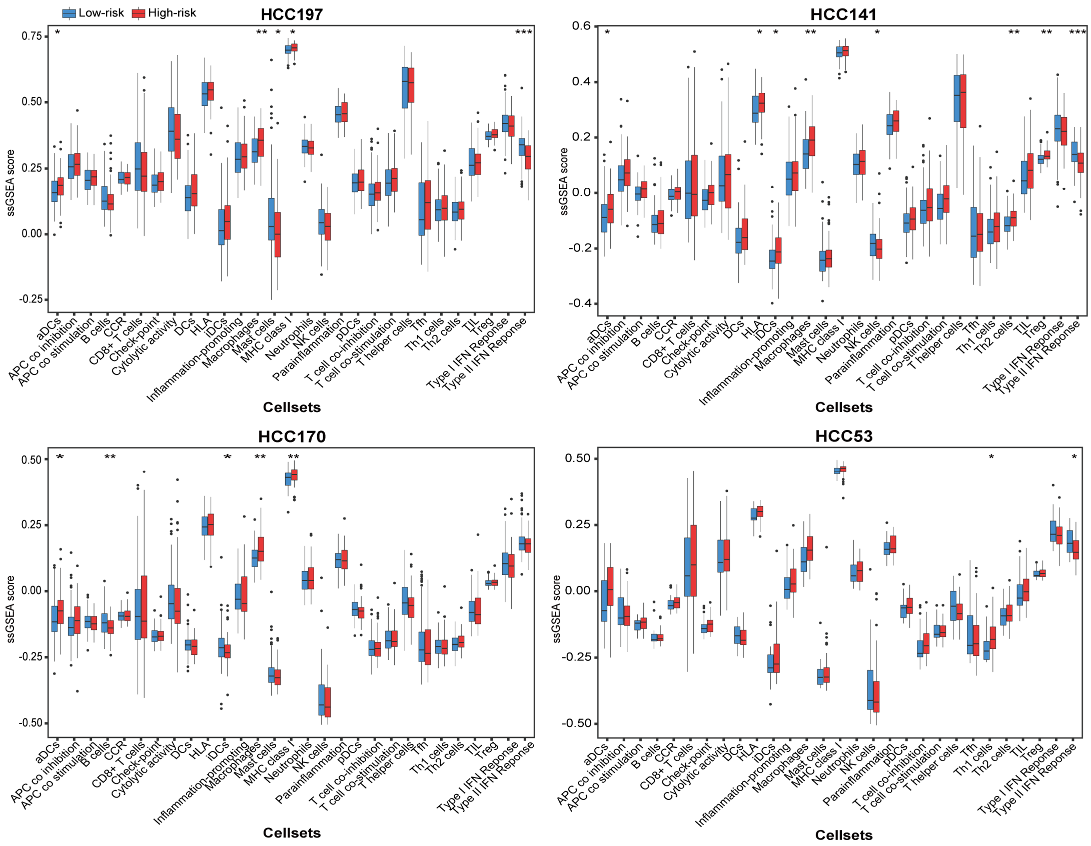

Figure S3. Distinct immune cell landscape for HCC prognostic groups. Box plots showed the comparison of ssGSEA score on the immune cell infiltrate levels between the high- and low-risk groups. Statistical significance was concluded at *p-value < 0.05, ** p-value < 0.01, *** p-value < 0.001.

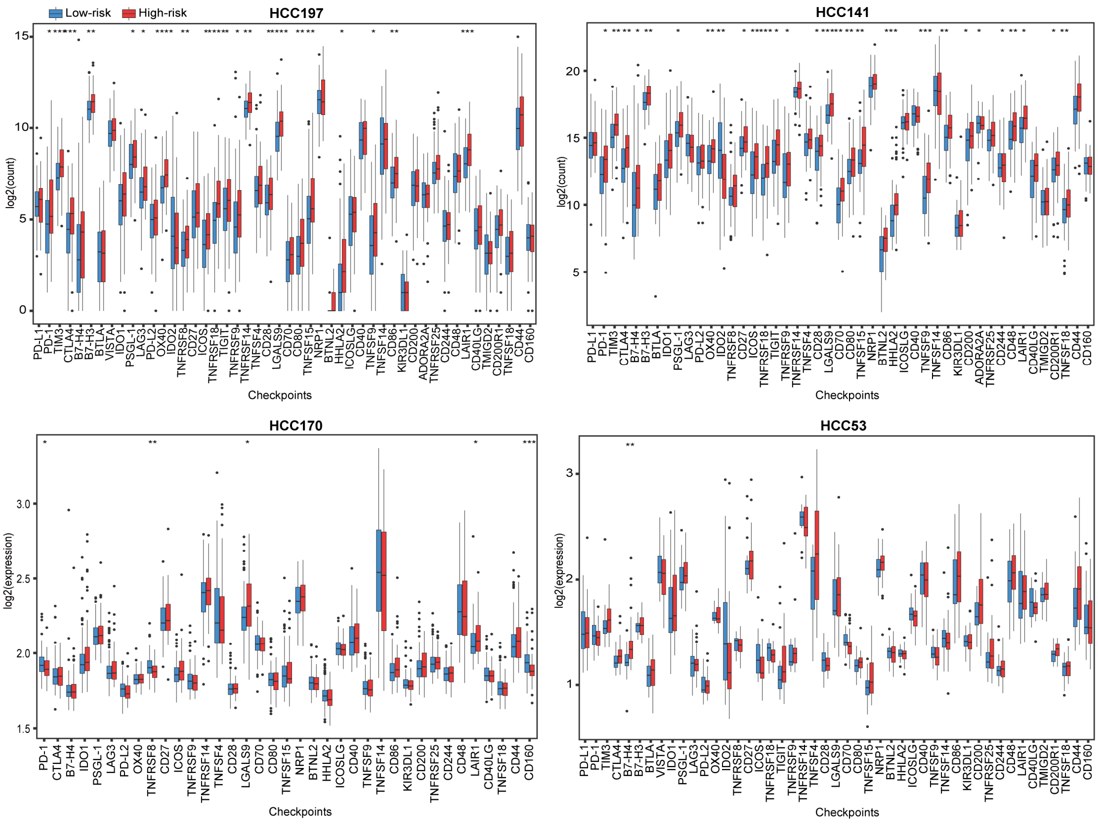

Figure S4. Distinct immune checkpoint genes for HCC prognostic groups. Box plots showed the ssGSEA score of the 48 immune checkpoint genes between high- and low-risk groups in four datasets. Statistical significance was concluded at *p-value < 0.05, ** p-value < 0.01, *** p-value < 0.001.

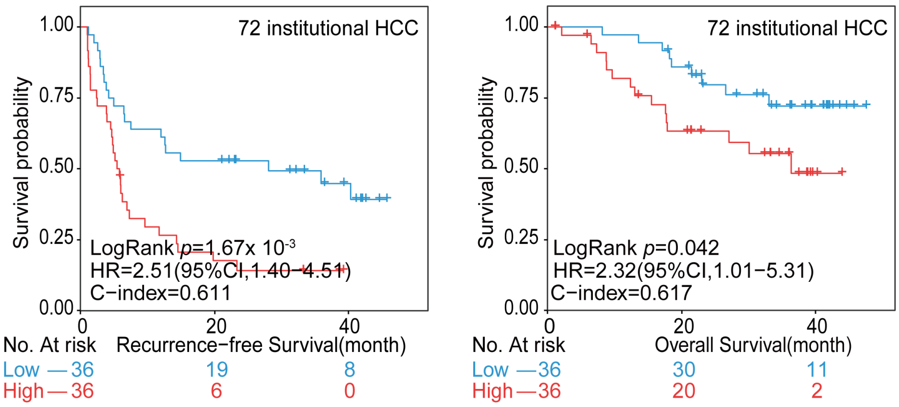

Figure S5. Validation of the signature in 72 institutional transcriptomic data. Kaplan-Meier curves of the RFS and OS for the 72 institutional transcriptomic data. According to the half-voting rule, patients were considered the high-risk group (red line) when at least five gene pairs suggested that this patient was at high risk; otherwise, the patients were included in the low-risk group (blue line).

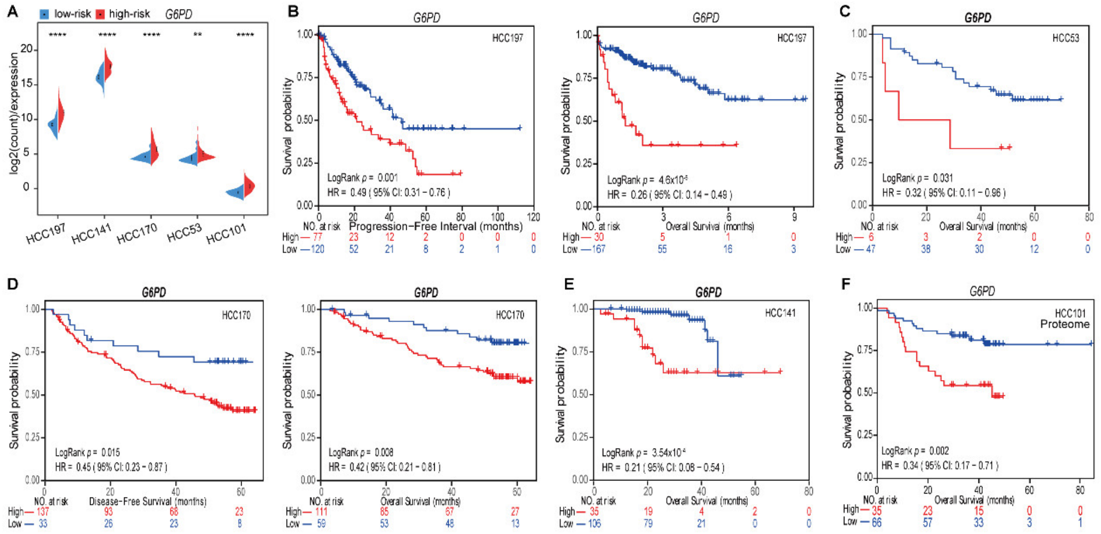

Figure S6. Analysis on G6PD. (A) The expression level of G6PD between the high- and low-risk groups. Statistical significance p-values were calculated by edgeR and Student's t-test, and the p values were labeled above each split violin plot with asterisks (*p-value < 0.05, ** p-value < 0.01, ***p-value < 0.001, **** p-value < 0.0001). The Kaplan-Meier curves for two prognostic groups were predicted by the optimal cut-off expression of G6PD in four transcriptomic data (B-E) and the proteomic data HCC101 (F).

Table S1. A list of 2,939 metabolism-related genes used in this study.

Classification	Gene Symbol
ABC Transporter	ABCA1,ABCA10,ABCA12,ABCA13,ABCA2,ABCA3,ABCA4,ABCA5,ABCA6,ABCA7,ABCA8,ABCA9,ABCB1,ABCB10,ABCB11,ABCB4,ABCB5,ABCB6,ABCB7,ABCB8,ABCB9,ABCC1,ABCC10,ABCC11,ABCC12,ABCC13,ABCC2,ABCC3,ABCC4,ABCC5,ABCC6,ABCC8,ABCC9,ABCD1,ABCD2,ABCD3,ABCD4,ABCE1,ABCF1,ABCF2,ABCF3,ABCG1,ABCG2,ABCG4,ABCG5,ABCG8
Amino Acid	AADAT,ACY1,ACY1L,ACY3,ADC,ADI1,AGPS,AGXT,AGXT2,AGXT2L1,AGXT2L2,ASNS,ASNSD1,ASPA,ASPG,ASRGL1,CCBL1,CCBL2,CRYM,DAO,DDO,GCAT,GCDH,GPT,GPT2,ILVBL,LGSN,PCBD1,PCBD2,SCCPDH,SCLY,SDS,SDSL,SEPHS1,SEPHS2,THNSL1,THNSL2,TMLHE
Aminosugar	CHI3L1,CHI3L2,CHIA,CHID1,CHIT1,CMAS,GFPT1,GFPT2,GNE,GNPDA1,GNPDA2,GNPNAT1,GNPTAB,GNPTG,NAGK,NANP,NANS,NPL,PGM3,RENBP,UAP1,UAP1L1
Anaerobic Glycolysis	LDHA,LDHAL6A,LDHAL6B,LDHB,LDHC,LDHD
ATPase	ATP13A1,ATP13A2,ATP13A3,ATP13A4,ATP13A5
B12	MMAB,MMACHC,MMADHC,MTRR
BCAAs	BCAT1,BCAT2,BCKDHA,BCKDHB,DBT,HIBADH,HIBCH,IVD,MCCC1,MCCC2
BH4	GCH1,PTS,QDPR,SPR
Bile Acid	BAAT,CYP27A1,CYP7A1,CYP7B1,CYP8B1,HSD3B7
Carbohydrate Storage	FBP1,FBP2,G6PC,G6PC2,G6PC3,GBE1,GYG1,GYG2,GYS1,GYS2,PCK1,PCK2,UGP2
Cholesterol	CYP51A1,DHCR24,DHCR7,EBP,EBPL,FDFT1,FDPS,HSD17B7,LBR,LCAT,LSS,NSDHL,SC4MOL,SC5DL,SQLE,TM7SF2
CoA	AACS,ACOT1,ACOT11,ACOT12,ACOT2,ACOT4,ACOT6,ACOT7,ACOT8,ACOT9,COASY,PANK1,PANK2,PANK3,PANK4,PPCDC,PPCS
Cofactor	AHCY,AHCYL1,AHCYL2,BBOX1,BTD,CEL,CRAT,CUBN,CYP24A1,CYP27B1,DAK,FLAD1,FOLH1,GPHN,HLCS,MOCOS,MOCS1,MOCS2,MOCS3,NAGS,RFK,THTPA,TPK1,VKORC1,VKORC1L1
Complex I	MT-ND1,MT-ND2,MT-ND3,MT-ND4,MT-ND4L,MT-ND5,MT-ND6,NDUFA1,NDUFA10,NDUFA11,NDUFA12,NDUFA13,NDUFA2,NDUFA3,NDUFA4,NDUFA4L,NDUFA4L2,NDUFA5,NDUFA6,NDUFA7,NDUFA8,NDUFA9,NDUFAB1,NDUFAF1,NDUFAF2,NDUFB1,NDUFB10,NDUFB11,NDUFB2,NDUFB3,NDUFB4,NDUFB5,NDUFB6,NDUFB7,NDUFB8,NDUFB9,NDUFC1,NDUFC2,NDUFS1,NDUFS2,NDUFS3,NDUFS4,NDUFS5,NDUFS6,NDUFS7,NDUFS8,NDUFV1,NDUFV2,NDUFV3
Complex II	SDHA,SDHB,SDHC,SDHD
Complex III	CYC1,CYCS,MT-CYB,UCRC,UQCR,UQCRB,UQCRC1,UQCRC2,UQCRFS1,UQCRH,UQCRQ
Complex IV	COX1,COX10,COX11,COX15,COX16,COX17,COX18,COX19,COX2,COX3,COX4I1,COX4I2,COX5A,COX5B,COX6A1,COX6A2,COX6B1,COX6B2,COX6C,COX7A1,COX7A2,COX7A2L,COX7B,COX7B2,COX7C,COX8A,COX8C
Creatine	CKB,CKBE,CKM,CKMT1A ,CKMT1B,CKMT2,GAMT,GATM
Cysteine	ADO,CBS,CDO1,CSAD,CTH,NFS1,PCYOX1,PCYOX1L,SUOX
Detox	AADAC,AADACL1,AADACL2,AADACL3,AADACL4,AKR7L,BCHE,CAT,CES1,CES2,CES3,CES7,DHDH,DPEP1,DPEP2,DPEP3,EPHX1,EPHX2,FMO1,FMO2,FMO3,FMO4,FMO5,SOD1,SOD2,SOD3,TPMT,UGT1A

	1,UGT1A10,UGT1A3,UGT1A4,UGT1A5,UGT1A6,UGT1A7,UGT1A8,UGT1A9,UGT2A1,UGT2A2,UGT2A3,UGT2B10,UGT2B11,UGT2B15,UGT2B17,UGT2B28,UGT2B4,UGT2B7,UGT3A1,UGT3A2
Fatty Acid	ACAA1,ACAA2,ACACA,ACACB,ACAD10,ACAD11,ACAD8,ACAD9,ACADL,ACADM,ACADS,ACADSB,ACADVL,ACAT1,ACAT2,ACBD3,ACBD4,ACBD5,ACBD6,ACBD7,ACOX1,ACOX2,ACOX3,ACOXL,ACSBG1,ACSBG2,ACSF2,ACSF3,ACSL1,ACSL3,ACSL4,ACSL5,ACSL6,ACSM1,ACSM2A,ACSM2B,ACSM3,ACSM4,ACSM5,ACSS1,ACSS2,ACSS3,CLYBL,CPT1A,CPT1B,CPT1C,CPT2,CYP4A11,CYP4A22,CYP4B1,CYP4F11,CYP4F12,CYP4F2,CYP4F22,CYP4F3,CYP4F8,CYP4V2,CYP4X1,CYP4Z1,DCI,DECR1,DECR2,DGAT1,DGAT2,DGAT2L3,DGAT2L4,DGAT2L6,DGAT2L7,ECH1,ECHDC1,ECHDC2,ECHDC3,ECHS1,EHHADH,ELOVL1,ELOVL2,ELOVL3,ELOVL4,ELOVL5,ELOVL6,ELOVL7,ETFA,ETFB,ET-FDH,FA2H,FAAH,FAAH2,FADS1,FADS2,FADS3,FAR1,FAR2,FASN,GPSN2,HACL1,HADH,HADHA,HADHB,LIPC,LIPF,LIPG,LIPH,LIPI,LIPJ,LIPK,LIPM,LIPN,LPL,LPPR2,MCAT,MECR,MGLL,MLYCD,MTTP,OLAH,OXSM,PECI,PECR,PHYH,PHYHD1,PNLIP,PNLIPRP1,PNLIPRP2,PNLIPRP3,PNPLA1,PNPLA2,PNPLA3,PNPLA4,PNPLA5,PNPLA6,PNPLA7,PNPLA8,SCD,SCD5,SCP2,SDR16C6,SDR39U1,SDR42E1,SDR9C7
Folate	DHFR,DHFRL1,FPGS,MTHFD1,MTHFD1L,MTHFD2,MTHFD2L,MTHFR,MTHFS,MTH-FSD,SHMT1,SHMT2
Glutamate	ALDH18A1,ALDH5A1,GAD1,GAD2,GADL1,GLS,GLS2,GLUD1,GLUD2,GLUL,OAT
Glutathione	GCLC,GCLM,GPX1,GPX2,GPX3,GPX4,GPX5,GPX6,GPX7,GPX8,GSR,GSS,GSTA1,GSTA2,GSTA3,GSTA4,GSTA5,GSTCD,GSTK1,GSTM1,GSTM2,GSTM3,GSTM4,GSTM5,GSTO1,GSTO2,GSTP1,GSTT1,GSTT2,HAGH,HAGHL,MGST1,MGST2,MGST3
Glycan	A4GALT,A4GNT,ABO,ALG1,ALG10,ALG10B,ALG11,ALG12,ALG13,ALG14,ALG2,ALG3,ALG5,ALG6,ALG8,ALG9,B3GALNT1,B3GALNT2,B3GALT1,B3GALT2,B3GALT4,B3GALT5,B3GALT6,B3GALTL,B3GAT1,B3GAT2,B3GAT3,B3GNT1,B3GNT2,B3GNT3,B3GNT4,B3GNT5,B3GNT6,B3GNT7,B3GNT8,B3GNT9,B3GNTL1,B4GALNT1,B4GALNT2,B4GALNT3,B4GALNT4,B4GALT1,B4GALT2,B4GALT3,B4GALT4,B4GALT5,B4GALT6,B4GALT7,C1GALT1,CTBS,DAD1,DDOST,DHDDS,DOLK,DOLPP1,DPAGT1,FN3K,FN3KRP,FUT1,FUT10,FUT11,FUT2,FUT3,FUT4,FUT5,FUT6,FUT7,FUT8,FUT9,GALE,GALNT1,GALNT10,GALNT11,GALNT12,GALNT13,GALNT14,GALNT2,GALNT3,GALNT4,GALNT5,GALNT6,GALNT7,GALNT8,GALNT9,GALNTL1,GALNTL2,GALNTL4,GALNTL5,GALNTL6,GA-NAB,GANC,GBA,GBA2,GBA3,GBGT1,GCNT1,GCNT2,GCNT3,GCNT4,GCNT6,GCNT7,GCS1,GLT1D1,GLT25D1,GLT25D2,GLT6D1,GLT8D1,GLT8D2,GLT8D3,GLT8D4,GTDC1,GYLTL1B,HAS1,HAS2,HAS3,LARGE,LY-CAT,MFNG,MGAT1,MGAT2,MGAT3,MGAT4A,MGAT4B,MGAT4C,MGAT5,MGAT5B,NAGA,NAGPA,POFUT1,POFUT2,POMGNT1,POMT1,POMT2,ST3GAL1,ST3GAL2,ST3GAL3,ST3GAL4,ST3GAL5,ST3GAL6,ST6GAL1,ST6GAL2,ST6GALNAC1,ST6GALNAC2,ST6GALNAC3,ST6GALNAC4,ST6GAL-NAC5,ST6GALNAC6,ST8SIA1,ST8SIA2,ST8SIA3,ST8SIA4,ST8SIA5,ST8SIA6,WBSCR17,XYLT1,XYLT2
Glycan Anchor	DPM1,DPM2,DPM3,GPLD1,PIGA,PIGB,PIGC,PIGF,PIGG,PIGH,PIGK,PIGL,PIGM,PIGN,PIGO,PIGP,PIGQ,PIGS,PIGT,PIGU,PIGV,PIGW,PIGX,PIGY,PIGZ
Glycan Degradation	AGA,EDEM1,EDEM2,EDEM3,FLJ21865,FUCA1,FUCA2,GALNS,GLA,GLB1,GLB1L,GLB1L2,GLB1L3,GNS,GUSB,HEXA,HEXB,HEXDC,HYAL1,HYAL2,HYAL3,HYAL4,IDS,IDUA,LYG1,LYZ,LYZL1,LYZL2,LYZL4,LYZL6,MAN1A1,MAN1A2,MAN1B1,MAN1C1,MAN2A1,MAN2A2,MAN2B1,MAN2B2,MAN2C1,MANBA,MANBAL,MANEA,MANEAL,NAGLU,NEU1,NEU2,NEU3,NEU4
Glycan Sulfate	CHPF,CHPF2,CHST1,CHST10,CHST11,CHST12,CHST13,CHST14,CHST2,CHST3,CHST4,CHST5,CHST6,CHST7,CHST8,CHST9,CHSY1,CHSY3,CSGALNACT1,CSGAL-NACT2,GAL3ST1,GAL3ST2,GAL3ST3,GAL3ST4,SGSH,SULF1,SULF2,UST
Glycine	AMT,BHMT,BHMT2,DMGDH,GCSH,GLDC,SARDH
Glycolysis	ADPGK,ALDOA,ALDOB,ALDOC,BPGM,DLAT,ENO1,ENO2,ENO3,GAPDH,GAPDHS,GCK,GPI,HK1,HK2,HK3,HKDC1,PDHA1,PDHA2,PDHB,PDHX,PFKFB1,PFKFB2,PFKFB3,PFKFB4,PFKL,PFKM,PFKP,PGAM1,PGAM2,PGAM4,PGAM5,PGK1,PGK2,PGM1,PGM2,PGM2L1,PGM5,PKLR,PKM2,TPI1
Glyoxylate	GLO1,GLOD4,GLOD5,HAO1,HAO2
Heparin Sulfate	EXT1,EXT2,EXT3,EXTL1,EXTL2,EXTL3,GLCE,HGSNAT,HPSE,HPSE2,HS2ST1,HS3ST1,HS3ST2,HS3ST3A1,HS3ST3B1,HS3ST4,HS3ST5,HS3ST6,HS6ST1,HS6ST2,HS6ST3,NDST1,NDST2,NDST3,NDST4
Histidine	AMDHD1,AMDHD2,FTCD,HAL,HDC,HNMT,UROC1
Hormone	AANAT,ACE,ACE2,ALOX12,ALOX12B,ALOX15,ALOX15B,ALOX5,ALOX5AP,ALOXE3,AOF1,AOF2,ASMT,AS-MTL,CYP20A1,DIO1,DIO2,DIO3,HPGD,IYD,LNPEP,LTA4H,LTC4S,MME,MMEL1,PGDS,PTGDS,PTGES,PTGES2,PTGES3,PTGIS,PTGR1,PTGR2,PTGS1,PTGS2,TBXAS1,TPO
Inositol Phosphate	CDIPT,FIG4,IMPA1,IMPA2,IM-PAD1,INPP1,INPP4A,INPP4B,INPP5A,INPP5B,INPP5D,INPP5E,INPP5F,INPP5J,INPP5K,INPPL1,IP6K1,IP6K2,IP6K3,IPMK,IPPK,ISYNA1,ITPK1,ITPKA,ITPKB,IT-PKC,MINPP1,MIOX,PGAP1,PI4K2A,PI4K2B,PI4KA,PI4KB,PIK3C2A,PIK3C2B,PIK3C2G,PIK3C3,PIK3CA,PIK3CB,PIK3CD,PIK3CG,PIK3R1,PIK3R2,PIK3R3,PIK3R4,PIK3R5,PIK3R6,PIP4K2A,PIP4K2B,PIP4K2C,PIP5K1A,PIP5K1B,PIP5K1C,PIP5K3,PIP5KL1,PTEN,SYNJ1,SYNJ2
Ion Transport	ATP11A,ATP11B,ATP11C,ATP12A,ATP1A1,ATP1A2,ATP1A3,ATP1A4,ATP1B1,ATP1B2,ATP1B3,ATP1B4,ATP2A1,ATP2A2,ATP2A3,ATP2B1,ATP2B2,ATP2B3,ATP2B4,ATP2C1,ATP2C2,ATP3,ATP4A,ATP4B,ATP7A,ATP7B,BEST1,BEST2,BEST3,BEST4,BSND,C14orf68,C20orf59,C6orf192,CACNA1A,CACNA1B,CACNA1C,CACNA1D,CACNA1E,CACNA1F,CACNA1G,CACNA1H,CACNA1I,CACNA1S,CACNA2D1,CACNA2D2,CACNA2D3,CACNA2D4,CACNB1,CACNB2,CACNB3,CACNB4,CACNG1,CACNG2,CACNG3,CACNG4,CACNG5,CACNG6,CACNG7,CACNG8,CATSPER1,CATSPER2,CATSPER3,CATSPERB,CFTR,CHRNA1,CHRNA10,CHRNA2,CHRNA3,CHRNA4,CHRNA5,CHRNA6,CHRNA7,CHRNA9,CHRNB1,CHRNB2,CHRNB3,CHRNB4,CHRND,CHRNE,CHRNG,CLCA1,CLCA2,CLCA3,CLCA4,CLCC1,CLCN1,CLCN2,CLC

	N3,CLCN4,CLCN5,CLCN6,CLCN7,CLCNKA,CLCNKB,CLIC1,CLIC2,CLIC3,CLIC4,CLIC5,CLIC6,CNGA1,CNGA2,CNGA3,CNGA4,CNGB1,CNGB3,GABRA1,GABRA2,GABRA3,GABRA4,GABRA5,GABRA6,GABRB1,GABRB2,GABRB3,GABRD,GABRE,GABRG1,GABRG2,GABRG3,GABRP,GABRQ,GABRR1,GABRR2,GABRR3,GLRA1,GLRA2,GLRA3,GLRA4,GLRB,GRIA1,GRIA2,GRIA3,GRIA4,GRID1,GRID2,GRIK1,GRIK2,GRIK3,GRIK4,GRIK5,GRIN1,GRIN2A,GRIN2B,GRIN2C,GRIN2D,GRIN3A,GRIN3B,HCN1,HCN2,HCN3,HCN4,HTR3A,HTR3B,HTR3C,HTR3D,HTR3E,ITPR1,ITPR2,ITPR3,KCNA1,KCNA10,KCNA2,KCNA3,KCNA4,KCNA5,KCNA6,KCNA7,KCNAB1,KCNAB2,KCNAB3,KCNB1,KCNB2,KCNC1,KCNC2,KCNC3,KCNC4,KCND1,KCND2,KCND3,KCNE1,KCNE1L,KCNE2,KCNE3,KCNE4,KCNF1,KCNG1,KCNG2,KCNG3,KCNG4,KCNH1,KCNH2,KCNH3,KCNH4,KCNH5,KCNH6,KCNH7,KCNH8,KCNJ1,KCNJ10,KCNJ11,KCNJ12,KCNJ13,KCNJ14,KCNJ15,KCNJ16,KCNJ2,KCNJ3,KCNJ4,KCNJ5,KCNJ6,KCNJ8,KCNJ9,KCNK1,KCNK10,KCNK12,KCNK13,KCNK15,KCNK16,KCNK17,KCNK18,KCNK2,KCNK3,KCNK4,KCNK5,KCNK6,KCNK7,KCNK9,KCNMA1,KCNMB1,KCNMB2,KCNMB3,KCNMB4,KCNN1,KCNN2,KCNN3,KCNN4,KCNQ1,KCNQ2,KCNQ3,KCNQ4,KCNQ5,KCNS1,KCNS2,KCNS3,KCNT1,KCNT2,KCNU1,KCNV1,KCNV2,KCTD1,KCTD10,KCTD11,KCTD12,KCTD13,KCTD14,KCTD15,KCTD16,KCTD17,KCTD18,KCTD19,KCTD2,KCTD20,KCTD21,KCTD3,KCTD4,KCTD5,KCTD6,KCTD7,KCTD8,KCTD9,P2RX1,P2RX2,P2RX3,P2RX4,P2RX5,P2RX6,P2RX7,SCN10A,SCN11A,SCN1A,SCN1B,SCN2A,SCN2B,SCN3A,SCN3B,SCN4A,SCN4B,SCN5A,SCN7A,SCN8A,SCN9A,SCNN1A,SCNN1B,SCNN1D,SCNN1G,SLC11A1,SLC11A2,SLC12A1,SLC12A2,SLC12A3,SLC12A4,SLC12A5,SLC12A6,SLC12A7,SLC12A8,SLC12A9,SLC13A1,SLC13A2,SLC13A3,SLC13A4,SLC13A5,SLC24A1,SLC24A2,SLC24A3,SLC24A4,SLC24A5,SLC24A6,SLC30A1,SLC30A10,SLC30A2,SLC30A3,SLC30A4,SLC30A5,SLC30A6,SLC30A7,SLC30A8,SLC30A9,SLC39A1,SLC39A10,SLC39A11,SLC39A12,SLC39A13,SLC39A14,SLC39A2,SLC39A3,SLC39A4,SLC39A5,SLC39A6,SLC39A7,SLC39A8,SLC39A9,SLC3A1,SLC3A2,SLC40A1,SLC41A1,SLC41A2,SLC41A3,VDAC1,VDAC2,VDAC3,ZACN,HEPH,LGICZ1,LST-3TM12,MAGT1,MCOLN1,MCOLN2,MCOLN3,MMAA,NALCN,NIPA1,OCA2,ORAI1,ORAI2,ORAI3,PKD1,PKD2,PKD2L1,RYR1,RYR2,RYR3,SLC22A24,SLC25A25,SLC31A1,SLC31A2,SLN,TCN1,TCN2,TF,TRPA1,TRPC1,TRPC3,TRPC4,TRPC5,TRPC6,TRPC7,TRPM1,TRPM2,TRPM3,TRPM4,TRPM5,TRPM6,TRPM7,TRPM8,TRPV1,TRPV2,TRPV3,TRPV4,TRPV5,TRPV6,TTYH1,TTYH2,TTYH3
Ketone Bodies	BDH1,BDH2,OXCT1,OXCT2
Krebs	ACLY,ACO1,ACO2,CS,D2HGDH,DLST ,FH,HCCS,IDH1,IDH2,IDH3A,IDH3B,IDH3G,L2HGDH,MDH1,MDH1B,MDH2,ME1,ME2,ME3,OGDH,OGDHL,PC,SUCLA2,SUCLG1,SUCLG2
Lysine	AASDH,AASDHPPT,AASS,PIPOX
Membrane Lipid	AGPAT1,AGPAT2,AGPAT3,AGPAT4,AGPAT5,AGPAT6,AGPAT9,CDS1,CDS2,CEPT1,CHKA,CHKB ,CHPT1,CLC,CRLS1,CROT,DAGLA,DAGLB,DGKA,DGKB,DGKD,DGKE,DGKG,DGKH,DGKI,DGKK,DGKQ,DGKZ,ETNK1,ETNK2,GDE1,GK,GK2,GK5,GNPAT,GPAM,GPD1,GPD1L,GPD2,LPCAT1,LPCAT2,LPCAT3,LPCAT4,LPGAT1,LPIN1,LPIN2,LPIN3,LYPLA1,LYPLA2,LY-PLAL1,MBOAT1,MBOAT2,MBOAT4,MBOAT7,MOGAT1,MOGAT2,MOGAT3,NAPE-PLD,PAP2D,PCYT1A,PCYT1B,PCYT2,PEMT,PGS1,PHOS-PHO1,PISD,PLA1A,PLA2G10,PLA2G12A,PLA2G12B,PLA2G15,PLA2G16,PLA2G1B,PLA2G2A,PLA2G2C,PLA2G2D,PLA2G2E,PLA2G2F,PLA2G3,PLA2G4A,PLA2G4B,PLA2G4C,PLA2G4D,PLA2G4E,PLA2G4F,PLA2G5,PLA2G6,PLA2G7,PLB1,PLCB1,PLCB2,PLCB3,PLCB4,PLCD1,PLCD3,PLCD4,PLCE1,PLCG1,PLCG2,PLCH1,PLCH2,PLCL1,PLCL2,PLCXD1,PLCXD2,PLCXD3,PLCZ1,PLD1,PLD2,PLD3,PLD4,PLD5,PLD6,PPAP2A,PPAP2B,PPAP2C,PPAPDC1A,PPAPDC1B,PPAPDC2,PPAPDC3,PRG2,PTDSS1,PTDSS2
Methionine	MAT1A,MAT2A,MAT2B,MSRA,MSRB2,MSRB3,MTR,SEPX1
Mevalonate	HMGCR,HMGCS1,HMGCS2,IDI1,IDI2,MVD,MVK,PMVK
Multipurpose	ABHD1,ABHD10,ABHD11,ABHD12,ABHD12B,ABHD13,ABHD14A,ABHD14B,ABHD2,ABHD3,ABHD4,ABHD5,ABHD6,ABHD7,ABHD8,ABHD9,ABP1,ACYP1,ACYP2,ADH1A,ADH1B,ADH1C,ADH4,ADH5,ADH6,ADH7,ADHFE1,AKR1A1,AKR1B1,AKR1B10,AKR1C1,AKR1C2,AKR1C3,AKR1CL2,AKR7A2,AKR7A3,ALDH16A1,ALDH1A1,ALDH1A2,ALDH1A3,ALDH1B1,ALDH1L1,ALDH1L2,ALDH2,ALDH3A1,ALDH3A2,ALDH3B1,ALDH3B2,ALDH6A1,ALDH7A1,ALDH8A1,ALDH9A1,AOC2,AOC3,ARSA,ARSB,ARSD,ARSE,ARSF,ARSG,ARSH,ARSI,ARSJ,ARSK,AUH,CA1,CA10,CA11,CA12,CA13,CA14,CA2,CA3,CA4,CA5A,CA5B,CA6,CA7,CA8,CA9,CBR1,CBR3,CBR4,DLD,GGH,GGT1,GGT2,GGT5,GGT6,GGT7,GGTLC1,GGTLC2,GOT1,GOT1L1,GOT2,GSTZ1,HMGCL,HMGCLL1,LPPR4,MAK10,MAOA,MAOB,NAT1,NAT10,NAT11,NAT12,NAT13,NAT14,NAT15,NAT2,NAT5,NAT6,NAT8,NAT8B,NAT8L,NAT9,POR,ZADH2
NAD	BST1,C9ORF95,DUOX1,DUOX2,NADK,NADSYN1,NAPRT1,NMNAT1,NMNAT2,NMNAT3,NNMT,NNT,NOX1,NOX3,NOX4,NOX5,NQO1,NQO2,QPRT
Neurotransmitter	ABAT,ACHE,CHAT,CHDH,COMT,COMTD1,DBH,DDC,NAALAD2,NAALADL1,NAALADL2,PNMT,SRR,TH,TPH1,TPH2
Nucleotide	BPNT1,CANT1,DCK,ENPP1,ENPP2,ENPP3,ENPP4,ENPP5,ENPP6,ENPP7,ENTPD1,ENTPD2,ENTPD3,ENTPD4,ENTPD5,ENTPD6,ENTPD7,ENTPD8,ITPA,NME1,NME2,NME3,NME4,NME5,NME6,NME7,NT5C,NT5C1A,NT5C1B,NT5C2,NT5C3,NT5C3L,NT5DC1,NT5DC2,NT5DC3,NT5DC4,NT5E,NT5M,NUDT1,NUDT10,NUDT11,NUDT12,NUDT13,NUDT14,NUDT15,NUDT16,NUDT16L1,NUDT17,NUDT18,NUDT19,NUDT2,NUDT21,NUDT22,NUDT3,NUDT4,NUDT5,NUDT6,NUDT7,NUDT8,NUDT9,RRM1,RRM2,RRM2B
Nucleotide Sugar	TGDS,UGDH,UXS1
Other	A1CF,ACN9,AOAH,APEH,AS3MT,BPHL,CMBL,CNDP1,CNDP2,DDHD1,DDHD2,ENOPH1,ENOSF1,EPX,ESD,FXN,GDPD1,GDPD2,GDPD3,GDPD4,GDPD5,GLYAT,GLYATL1,GLYATL2,GNMT,HISPPD1,HIS-PPD2A,IAH1,LIAS,LIPT1,MOSC1,MOSC2,MPO,NIT1,NIT2,PAM,PON1,PON2,PON3,PPA1,PPA2,PTER,THEM2,THEM4,THEM5,UEVLD,WWOX,GLTP

Other Transport	CTNS,DIRC2,FLVCR1,FLVCR2,MCART1,MCART2,MCART6,MFSD1,MFSD10,MFSD11,MFSD2,MFSD3,MFSD4,MFSD5,MFSD7,MFSD8,MFSD9,OSTalpha,OSTbeta,STRA6,TAP1,TAP2,TMCO3,TMEM104,TPCN1,TPCN2
Pentose Phosphate	DERA,G6PD,PGD,PGLS,RPE,RPIA,TALDO1,TKT,TKTL1,TKTL2
Pigment	DCT,DDT,TYR,TYRP1
Polyamine	AMACR,AMD1,MTAP,ODC1,PAOX,SAT1,SAT2,SATL1,SMOX,SMS,SRM
Porphyrin	ALAD,ALAS1,ALAS2,BLVRA,BLVRB,CPOX,FECH,HMBS,HMOX1,HMOX2,PPOX,UROD,UROS
Proline	ALDH4A1,PRODH,PRODH2,PYCR1,PYCR2,PYCRL
Propanoate	MCEE,MUT,PCCA,PCCB
Proton Transport	ATP5A1,ATP5B,ATP5C1,ATP5C2,ATP5D,ATP5E,ATP5F1,ATP5G1,ATP5G2,ATP5G3,ATP5H,ATP5I,ATP5J,ATP5J2,ATP5L,ATP5L2,ATP5O,ATP5S,ATP5SL,ATP6,ATP6AP1,ATP6AP2,ATP6V0A1,ATP6V0A2,ATP6V0A4,ATP6V0B,ATP6V0C,ATP6V0D1,ATP6V0D2,ATP6V0E1,ATP6V0E2,ATP6V1A,ATP6V1B1,ATP6V1B2,ATP6V1C1,ATP6V1C2,ATP6V1D,ATP6V1E1,ATP6V1E2,ATP6V1F,ATP6V1G1,ATP6V1G2,ATP6V1G3,ATP6V1H,HVCN1,TCIRG1,UCP1,UCP2,UCP3
Purine	ADA,ADAD1,ADAD2,ADAL,ADK,ADSL,ADSS,ADSSL1,AK1,AK2,AK3,AK3L1,AK5,AK7,AMPD1,AMPD2,AMPD3,APRT,ATIC,CECR1,DGUOK,FHIT,GART,GDA,GMPR,GMPR2,GMPS,GUK1,HPRT1,IMPDH1,IMPDH2,NP,PAICS,PFAS,PPAT,PRPS1,PRPS1L1,PRPS2,PRPSAP1,PRPSAP2,XDH
Pyrimidine	CAD,CDA,CDADC1,CMPK,CTPS,CTPS2,DCTD,DHODH,DPYD,DPYS,DPYSL2,DPYSL3,DPYSL4,DPYSL5,DTYMK,DUT,TK1,TK2,TYMP,TYMS,UCK1,UCK2,UCKL1,UMPS,UPB1,UPP1,UPP2,UPRT
Reactive Oxygen	AOX1,DDAH1,DDAH2,NOS1,NOS2,NOS3
Redox	CP,CRYZ,CRYZL1,CYB561,CYB5A,CYB5B,CYB5D1,CYB5D2,CYB5R1,CYB5R2,CYB5R3,CYB5R4,CYB5RL,CYBA,CYBASC3,CYBB,CYBRD1,DHRS1,DHRS11,DHRS12,DHRS13,DHRS2,DHRS3,DHRS4,DHRS4L1,DHRS4L2,DHRS7,DHRS7B,DHRS7C,DHRS9,DHRSX,DHTKD1,ENOX1,ENOX2,FDX1,FDX1L,FDXR,FOXRED1,FOXRED2,FRRS1,GLRX,GLRX2,GLRX3,GLRX5,GRXCR1,GRXCR2,ISCA1,ISCA1L,ISCA2,LPO,MICAL1,MICAL2,MICAL3,NDOR1,NMRAL1,OGFOD1,OGFOD2,OXA1L,PRDX1,PRDX2,PRDX3,PRDX4,PRDX5,PRDX6,PYROXD1,TXNRD1,TXNRD2,TXNRD3
Serine	PHGDH,PSAT,PSPH
Signalling	ADCY1,ADCY10,ADCY2,ADCY3,ADCY4,ADCY5,ADCY6,ADCY7,ADCY8,ADCY9,C9ORF98,CNP,GUCY1A2,GUCY1A3,GUCY1B3,GUCY2C,GUCY2D,GUCY2F,NPR1,NPR2,NPR3,OAS1,OAS2,OAS3,OASL,PDE10A,PDE11A,PDE12,PDE1A,PDE1B,PDE1C,PDE2A,PDE3A,PDE3B,PDE4A,PDE4B,PDE4C,PDE4D,PDE5A,PDE6A,PDE6B,PDE6C,PDE6D,PDE6G,PDE6H,PDE7A,PDE7B,PDE8A,PDE8B,PDE9A
Small Molecule Transport	ACCN1,ACCN2,ACCN3,ACCN4,ACCN5,ANKH,AQP1,AQP10,AQP11,AQP12A,AQP12B,AQP2,AQP3,AQP4,AQP5,AQP6,AQP7,AQP8,AQP9,ASNA1,ATP10A,ATP10B,ATP10D,ATP8,ATP8A1,ATP8A2,ATP8B1,ATP8B2,ATP8B3,ATP8B4,ATP9A,ATP9B,FOLR1,FOLR2,FOLR3,SLC10A1,SLC10A2,SLC10A3,SLC10A4,SLC10A5,SLC10A6,SLC10A7,SLC14A1,SLC14A2,SLC15A1,SLC15A2,SLC15A3,SLC15A4,SLC16A1,SLC16A10,SLC16A11,SLC16A12,SLC16A13,SLC16A14,SLC16A2,SLC16A3,SLC16A4,SLC16A5,SLC16A6,SLC16A7,SLC16A8,SLC16A9,SLC17A1,SLC17A2,SLC17A3,SLC17A4,SLC17A5,SLC17A6,SLC17A7,SLC17A8,SLC18A1,SLC18A2,SLC18A3,SLC19A1,SLC19A2,SLC19A3,SLC1A1,SLC1A2,SLC1A3,SLC1A4,SLC1A5,SLC1A6,SLC1A7,SLC20A1,SLC20A2,SLC22A1,SLC22A11,SLC22A12,SLC22A13,SLC22A14,SLC22A15,SLC22A16,SLC22A17,SLC22A18,SLC22A18AS,SLC22A2,SLC22A20,SLC22A23,SLC22A3,SLC22A4,SLC22A5,SLC22A6,SLC22A7,SLC22A8,SLC22A9,SLC23A1,SLC23A2,SLC23A3,SLC25A1,SLC25A10,SLC25A11,SLC25A12,SLC25A13,SLC25A14,SLC25A15,SLC25A16,SLC25A17,SLC25A18,SLC25A19,SLC25A2,SLC25A20,SLC25A21,SLC25A22,SLC25A23,SLC25A24,SLC25A25,SLC25A26,SLC25A27,SLC25A28,SLC25A29,SLC25A3,SLC25A30,SLC25A31,SLC25A32,SLC25A33,SLC25A34,SLC25A35,SLC25A36,SLC25A37,SLC25A38,SLC25A39,SLC25A4,SLC25A40,SLC25A41,SLC25A42,SLC25A43,SLC25A44,SLC25A45,SLC25A46,SLC25A5,SLC25A6,SLC26A1,SLC26A10,SLC26A11,SLC26A2,SLC26A3,SLC26A4,SLC26A5,SLC26A6,SLC26A7,SLC26A8,SLC26A9,SLC27A1,SLC27A2,SLC27A3,SLC27A4,SLC27A5,SLC27A6,SLC28A1,SLC28A2,SLC28A3,SLC29A1,SLC29A2,SLC29A3,SLC29A4,SLC2A1,SLC2A10,SLC2A11,SLC2A12,SLC2A13,SLC2A14,SLC2A2,SLC2A3,SLC2A4,SLC2A5,SLC2A6,SLC2A7,SLC2A8,SLC2A9,SLC32A1,SLC33A1,SLC34A1,SLC34A2,SLC34A3,SLC35A1,SLC35A2,SLC35A3,SLC35A4,SLC35A5,SLC35B1,SLC35B2,SLC35B3,SLC35B4,SLC35C1,SLC35C2,SLC35D1,SLC35D2,SLC35D3,SLC35E1,SLC35E2,SLC35E3,SLC35E4,SLC35F1,SLC35F2,SLC35F3,SLC35F4,SLC35F5,SLC36A1,SLC36A2,SLC36A3,SLC36A4,SLC37A1,SLC37A2,SLC37A3,SLC37A4,SLC38A1,SLC38A2,SLC38A3,SLC38A4,SLC38A5,SLC38A6,SLC38A7,SLC43A1,SLC43A2,SLC43A3,SLC44A1,SLC44A2,SLC44A3,SLC44A4,SLC44A5,SLC45A1,SLC45A2,SLC45A3,SLC45A4,SLC46A1,SLC46A2,SLC46A3,SLC47A1,SLC47A2,SLC4A1,SLC4A10,SLC4A11,SLC4A1AP,SLC4A2,SLC4A3,SLC4A4,SLC4A5,SLC4A7,SLC4A8,SLC4A9,SLC5A1,SLC5A10,SLC5A11,SLC5A12,SLC5A2,SLC5A3,SLC5A4,SLC5A5,SLC5A6,SLC5A7,SLC5A8,SLC5A9,SLC6A1,SLC6A11,SLC6A12,SLC6A13,SLC6A14,SLC6A15,SLC6A16,SLC6A17,SLC6A18,SLC6A19,SLC6A2,SLC6A20,SLC6A3,SLC6A4,SLC6A5,SLC6A6,SLC6A7,SLC6A8,SLC6A9,SLC7A1,SLC7A10,SLC7A11,SLC7A13,SLC7A14,SLC7A2,SLC7A3,SLC7A4,SLC7A5,SLC7A6,SLC7A7,SLC7A8,SLC7A9,SLC8A1,SLC8A2,SLC8A3,SLC9A1,SLC9A10,SLC9A11,SLC9A2,SLC9A3,SLC9A4,SLC9A5,SLC9A6,SLC9A7,SLC9A8,SLC9A9,SLCO1A2,SLCO1B1,SLCO1B3,SLCO1C1,SLCO2A1,SLCO2B1,SLCO3A1,SLCO4A1,SLCO4C1,SLCO5A1,SLCO6A1,MIP,NPC1,NPC1L1,NPC2,PCTP,RHAG,RHBG,RHCG,SFXN1,SFXN2,SFXN3,SFXN4,SFXN5,SPNS1,STAR,STARD10,STARD13,STARD3,STARD3NL,STARD4,STARD5,STARD6,STARD7,STARD8,STARD9,SV2A,SV2B,SV2C,SVOP

Sphingolipid	CERK,CERKL,DEGS1,DEGS2,GALC,LASS1,LASS2,LASS3,LASS4,LASS5,LASS6,SGMS1,SGMS2,SGPL1,SGPP1,SGPP2,SMPD1,SMPD2,SMPD3,SMPD4,SMPDL3A,SMPDL3B,SPHK1,SPHK2,SPTLC1,SPTLC2,SPTLC3,UGCG,UGCGL1,UGCGL2,UGT8
Sphingosine	ACER1,ACER2,ACER3,ASAH1,ASAH2,ASAH2B,ASAH2C,ASAHL,KDSR
Steroid	AKR1C4,AKR1D1,CH25H,CYP11A1,CYP11B1,CYP11B2,CYP17A1,CYP19A1,CYP1A1,CYP1A2,CYP1B1,CYP21A2,CYP27C1,CYP2A13,CYP2A6,CYP2A7,CYP2B6,CYP2C18,CYP2C19,CYP2C8,CYP2C9,CYP2D6,CYP2E1,CYP2F1,CYP2J2,CYP2R1,CYP2S1,CYP2U1,CYP2W1,CYP39A1,CYP3A4,CYP3A43,CYP3A5,CYP3A7,CYP46A1,GGPS1,HSD11B1,HSD11B1L,HSD11B2,HSD17B1,HSD17B10,HSD17B11,HSD17B12,HSD17B13,HSD17B14,HSD17B2,HSD17B3,HSD17B4,HSD17B6,HSD17B8,HSD3B1,HSD3B2,HSDL1,HSDL2,KL,KLB,LIPA,LIPE,SOAT1,SOAT2,SRD5A1,SRD5A2,SRD5A2L2,SRD5A3
Sugar	AGK,AGL,AMY1A,AMY1B,AMY1C,AMY2A,AMY2B,C9ORF103,CRYL1,FPGT,FUK,GAA,GALK1,GALK2,GALM,GALT,GFOD1,GFOD2,GLYCTK,GMDS,GMPPA,GMPPB,GRHPR,H6PD,HYI,KHK,LALBA,LCT,LCTL,MGAM,MPI,PMM1,PMM2,PYGB,PYGL,PYGM,RBKS,SI,SIAE,SORD,TREH,TSTA3
Sulfate	DSE,DSEL,GALNAC4S-6ST,MPST,PAPSS1,PAPSS2,SRXN1,STS,SULT1A1,SULT1A2,SULT1A3,SULT1A4,SULT1B1,SULT1C2,SULT1C3,SULT1C4,SULT1E1,SULT2A1,SULT2B1,SULT4A1,SULT6B1,TST
Tryptophan	ACMSD,AFMID,HAAO,IDO1,IDO2,INMT,KMO,KYNU,TDO2
Tyrosine	FAH,FAHD1,FAHD2A,FAHD2B,HGD,HPD,HPDL,PAH,TAT
Ubiquinone	CABC1,COQ10A,COQ10B,COQ2,COQ3,COQ4,COQ5,COQ6,COQ7,COQ9,PDSS1,PDSS2,SQRDL
Urea	AGMAT,ARG1,ARG2,ASL,ASS1,CPS1,OTC
Vitamin A	BCMO1,BCO2,CYP26A1,CYP26B1,CYP26C1,LRAT,RDH10,RDH11,RDH12,RDH13,RDH14,RDH16,RDH5,RDH8,RETSAT,RPE65,SDR16C5
Vitamin B6	PDXK,PDXP,PHOSPHO2,PNPO
Xylulose	DCXR,XYLB
188 metabolic genes from the latest metabolic pathways in the Kyoto Encyclopaedia of Genes and Genomes	ALG13,OGT,A3GALT2,ASPDH,LIPT2,ADPRM,SELE-NOI,MGAM2,EOGT,MTM1,NTPCR,ACP5,ALPL,LOC107987478,IL4I1,SPAM1,MTMR1,ACP1,ALPI,EARS2,CYP2D7,MTMR2,ACP2,CMPK2,CBSL,CARNS1,c14orf159,GGCT,MTMR3,GPAT2,Alpp,CHAC1,LFNG,MTMR4,ALPPL2,NMRK2,RPEL1,PPT1,CHAC2,RFNG,MTMR8,ETHE1,PPT2,GGCX,OPLAH,MTMR6,TMEM189,STT3A,MTMR7,TMEM189-UBE2V1,HACD2,STT3B,MTMR14,VNN1,SELENBP1,HACD1,RPN1,VNN2,HACD4,NME1-NME2,LAP3,RPN2,MTFMT,NT5C1B-RDH14,VNN3,HACD3,AK9,ANPEP,HYKK,PSTK,TUSC3,POMGNT2,jmjd7-pla2g4b,ACSM6,LOC390877,MIF,SEPSECS,POGLUT1,SACM1L,mri1,MARS,POMK,NUS1,APIP,CAMKMT,LRTOMT,MARS2,ISPD,RIMKLB,KMT2A,XXYLT1,fktn,GPAA1,PGP,FNTB,FNTA,RIM-KLA,KMT2D,FKRP,RCE1,RGN,KMT2C,CARNMT1,TMEM5,ST20-MTHFS,ZMPSTE24,ABHD14A-ACY1,KMT2B,POC1B-GALNT4,PLOD3,OCRL,NAMPT,ICMT,DNMT1,KMT2E,CD38,GSTT2B,DNMT3A,SETD1B,C1GALT1C1,TIGAR,DNMT3B,SETD1A,C1GALT1C1L,SIRT1,SETD7,SIRT2,PRDM7,SIRT4,ENO4,prdm9,SIRT3,ASH1L,SIRT5,SMYD1,FAM213B,HOGA1,SIRT6,SMYD2,SIRT7,SUV39H1,SMYD3,MGAT4D,NADK2,SUV39H2,CYP3A7-CYP3A51P,EHMT2,EHMT1,SETDB1,SETDB2,TMEM86B,PRDM2,LOC102724788,EZH1,EZH2,LOC107987479,NSD1,whsc1,P4HA2,TXNDC12,whsc1l1,P4HA3,setmar,P4HA1,PAFAH1B1,PAFAH1B2,PAFAH1B3,NDUFC2-KCTD14,DCTPP1,SETD2,DOT1L,PRDM6,KMT5B,L3HYPDH,KMT5C,PAFAH2,UQCRHL,PLOD2,PLOD1,PRUNE1,HDDC3,GPCPD1,TAZ,AK6,LHPP,URAD,ALLC

Table S2. Eight genes were included the Periportal signature[1] .

Gene Name	Gene Symbol	Entrez Gene ID
glutaminase 2	GLS2	27165
fetuin B	FETUB	26998
solute carrier family 22 member 7	SLC22A7	10864
glycine N-methyltransferase	GNMT	27232
solute carrier family 10 member 1	SLC10A1	6554
alanine-glyoxylate aminotransferase	AGXT	189
ornithine carbamoyltransferase	OTC	5009
solute carrier family 27 member 5	SLC27A5	10998

Note: [1] from Désert., et al. (doi: 10.1002/hep.29254). .

Table S3. 43 Proliferation-associated genes[1.]

Gene Name	Gene Symbol	Entrez Gene ID
aurora kinase B	AURKB	9212
baculoviral IAP repeat containing 5	BIRC5	332
BUB1 mitotic checkpoint serine/threonine kinase	BUB1	699
cyclin A2	CCNA2	890
cyclin B1	CCNB1	891
cyclin E1	CCNE1	898
cyclin F	CCNF	899
cell division cycle 20	CDC20	991
cell division cycle 25C	CDC25C	995
cell division cycle 6	CDC6	990
cell division cycle 7	CDC7	8317
centromere protein E	CENPE	1062
centromere protein F	CENPF	1063
checkpoint kinase 1	CHEK1	1111
CDC28 protein kinase regulatory subunit 2	CKS2	1164
DEAD/H-box helicase 11	DDX11	1663
dihydrofolate reductase	DHFR	1719
DNA methyltransferase 1	DNMT1	1786
E2F transcription factor 3	E2F3	1871
exosome component 9	EXOSC9	5393
flap structure-specific endonuclease 1	FEN1	2237
MAD2 mitotic arrest deficient-like 1	MAD2L1	4085
mitogen-activated protein kinase 13	MAPK13	5603
minichromosome maintenance complex component 3	MCM3	4172
minichromosome maintenance complex component 4	MCM4	4173
minichromosome maintenance complex component 5	MCM5	4174
minichromosome maintenance complex component 6	MCM6	4175
marker of proliferation Ki-67	MKI67	4288
nuclear autoantigenic sperm protein	NASP	4678
origin recognition complex subunit 1	ORC1	4998
proliferating cell nuclear antigen	PCNA	5111
protein kinase, membrane associated tyrosine/threonine 1	PKMYT1	9088
polo like kinase 1	PLK1	5347
primase	PRIM1	5557
pituitary tumor-transforming 1	PTTG1	9232
replication factor C subunit 1	RFC1	5981
ribonucleotide reductase catalytic subunit M1	RRM1	6240
ribonucleotide reductase regulatory subunit M2	RRM2	6241
TIMP metallopeptidase inhibitor 1	TIMP1	7076
topoisomerase	TOP2A	7153
TRAF interacting protein	TRAIP	10293
thymidylate synthetase	TYMS	7298
uracil DNA glycosylase	UNG	7374
CTP synthase 1	CTPS1	1503
MYB proto-oncogene, transcription factor	MYB	4602

Note: 1 from Whitfield, Michael L. , et al. (doi: 10.1038/nrc1802). .

Table S4. 108 metabolism pathways [1] .

Pathway	logFC	t	P.Value	adj.P.Val	B	Gene
Alanine, Aspartate and Glutamate Metabolism - Homo sapiens (human)	-0.0008	-0.0139	0.9889	0.9889	-6.8725	CKMT1A,CKMT1B,CYCS,NME4,TAZ,TMEM256-PLSCR3
Arginine Biosynthesis - Homo sapiens (human)	-0.3492	-6.2158	2.83E-09	7.00E-08	10.7027	CRLS1,PGS1,PTPMT1,TAMM41
Cysteine and Methionine Metabolism - Homo sapiens (human)	0.2242	2.9759	0.0033	0.0051	-2.5633	ACAT2,CYP51A1,DHCR24,DHCR7,EBP,FDFT1,FDPS,GGPS1,HMGCR,HMGCS1,HSD17B7,IDI1,LBR,LIPA,LSS,MSMO1,MVD,MVK,NSDHL,PMVK,SC5D,SOAT1,SQLE,TM7SF2
Glycine, Serine and Threonine Metabolism - Homo sapiens (human)	0.3495	5.0533	9.62E-07	3.25E-06	5.0818	ACLY,ACO1,ACO2,CS,DLAT,DLD,DLST,FH,IDH1,IDH2,IDH3A,IDH3B,IDH3G,MDH1,MDH2,MPC1,OGDH,OGDHL,PC,PCK1,PCK2,PDHA1,PDHA2,PDHB,SDHA,SDHB,SDHC,SDHD,SUCLA2,SUCLG1,SUCLG2,PDHX
Histidine Metabolism - Homo sapiens (human)	0.2833	5.5213	1.02E-07	5.05E-07	7.2425	ANXA1,MKNK1,PLA2G4A,PTGS1,PTGS2,VIM
Lysine Degradation - Homo sapiens (human)	0.2311	3.5438	0.0005	0.0009	-0.8153	AKR1A1,AKR1B1,AKR1C3,CBR1,FAM213B,HPGDS,PTGDS,PTGES,PTGES2,PTGES3
Estradiol Biosynthesis - Homo sapiens (human)	-0.5380	-5.9797	9.85E-09	9.67E-08	9.4941	ADSL,ADSS,ADSSL1,AK1,AK2,AK3,AK4,AK5,ATIC,GART,GMPS,IMPDH1,IMPDH2,PAICS,PFAS,PPAT,PRPS1,PRPS1L1,PRPS2,TAF9
Phenylalanine, Tyrosine and Tryptophan Biosynthesis - Homo sapiens (human)	-0.5380	-5.9797	9.85E-09	9.67E-08	9.4941	CAD,CMPK1,CMPK2,CTPS1,CTPS2,DHODH,NME1,NME2,NME3,NME4,NME6,UMPS
Tryptophan Metabolism - Homo sapiens (human)	-0.5380	-5.9797	9.85E-09	9.67E-08	9.4941	DDC,QDPR,SLC18A2,TH
Tyrosine Metabolism - Homo sapiens (human)	-0.3670	-5.9027	1.47E-08	1.22E-07	9.1068	DBH,DDC,PNMT,QDPR,SLC18A2,TH
Valine, Leucine and Isoleucine Biosynthesis - Homo sapiens (human)	-0.6224	-2.5682	0.0107	0.0442	-3.5199	DBH,DDC,QDPR,SLC18A2,TH
Valine, Leucine and Isoleucine Degradation - Homo sapiens (human)	-0.5792	-7.1710	1.34E-11	1.45E-09	15.9005	ABCD1,ABCD2,ACAA2,ACAD9,ACADL,ACADM,ACADS,ACADVL,ACAT1,ACSL1,ACSL3,ACSL4,ACSL5,ACSL6,ACSM1,ACSM2A,ACSM2B,ACSM3,ACSM4,ACSS1,ACSS2,ACSS3,CPT1A,CPT1B,CPT2,CRAT,CROT,ECHS1,ETFA,ETFB,ETFDH,HADH,HADHA,HADHB,HSD17B10,PEX11G,PEX13,PEX14,SLC25A20,ACAT2,ACAA1,EHHADH,ACOX3,ACOX1,ACADSB,GCDH,ACSBG1,ACSBG2,CPT1C,ECI1,ECI2,CYP4A11,CYP4A22,ADH1A,ADH1B,ADH1C,ADH7,ADH4,ADH5,ADH6,ALDH2,ALDH3A2,ALDH1B1,ALDH7A1,ALDH9A1
Amino Sugar and Nucleotide Sugar Metabolism - Homo sapiens (human)	-0.2150	-4.5598	8.82E-06	2.27E-05	2.9663	ACACA,ACACB,ACLY,FASN,MCAT,OLAH,OXSM,ACAA2,ECHS1,HADHA,HADHB,HSD17B10,MECR,PPT1,HADH,PPT2,ELOVL1,ELOVL2,ELOVL3,ELOVL4,ELOVL5,ELOVL6,ELOVL7,HSD17B12,HACD2,HACD1,HACD4,HACD3,TECR,ACOT4,ACOT2,ACOT1,ACOT7
Butanoate Metabolism - Homo sapiens (human)	-0.3633	-5.3312	2.57E-07	1.03E-06	6.3474	ACACA,ACACB,MCAT,FASN,OXSM,OLAH,ACSL6,ACSL4,ACSL1,ACSL5,ACSL3,ACSBG1,ACSBG2
Galactose Metabolism - Homo sapiens (human)	-0.2059	-4.2699	3.00E-05	7.19E-05	1.8056	GCH1,ALPL,ALPI,ALPP,ALPPL2,DHFR,DHFR2,FPGS,GGH,PTS,SPR,QDPR,MOCS1,MOCS2

Inositol Phosphate Metabolism - Homo sapiens (human)	-0.1735	-3.7241	0.0003	0.0005	-0.2039	ACSS1,ACSS2,ADH1A,ADH1B,ADH1C,ADH4,ADH5,ADH6,ADH7,ADPGK,AKR1A1,ALDH1A3,ALDH1B1,ALDH2,ALDH3A1,ALDH3A2,ALDH3B1,ALDH3B2,ALDH7A1,ALDH9A1,ALDOA,ALDOB,ALDOC,BPGM,DLAT,DLD,ENO1,ENO2,ENO3,FBP1,FBP2,G6PC,G6PC2,GALM,GAPDH,GAPDHS,GCK,GPI,HK1,HK2,HK3,HKDC1,LDHA,LDHAL6A,LDHAL6B,LDHB,LDHC,PANK1,PCK1,PCK2,PDHA1,PDHA2,PDHB,PFKFB1,PFKFB2,PFKFB3,PFKFB4,PFKL,PFKM,PFKP,PGAM1,PGAM2,PGAM4,PGK1,PGK2,PGM1,PGM2,PKLR,PKM,SLC2A2,TPI1
Pentose and Glucuronate Interconversions - Homo sapiens (human)	-0.0132	-0.2973	0.7665	0.8037	-6.8286	GBE1,GCK,GSK3A,GSK3B,GYG1,GYS1,GYS2,PGM1,PGM2,PGM3,PPP1CA,PPP1CB,PPP1CC,PPP1R3A,PPP1R3B,PPP1R3C,PPP1R3D,PPP1R3E
Nitrogen Metabolism - Homo sapiens (human)	-0.0691	-1.5925	0.1128	0.1385	-5.6195	GFPT1,GNPNAT1,GPI,MGEA5,OGT,PGM3,UAP1
Sulfur Metabolism - Homo sapiens (human)	0.8032	2.4371	0.0153	0.0592	-3.8385	AHCY,CARM1,COMT,DOT1L,GAMT,HNMT,MRM2,MTHFR,NNMT,PCMT1,PEMT,PNMT,PRMT1,PRMT5,PRMT6,RNMT,SETDB1,SUV39H2
Glycosaminoglycan Degradation - Homo sapiens (human)	0.0077	0.1812	0.8564	0.8809	-6.8562	MTHFR,MTR,MTRR,AHCY,ALDH7A1,BHMT,CHDH,MAT2A,MAT2B,MTR,PEMT
Mucin Type O-Glycan Biosynthesis - Homo sapiens (human)	-0.3943	-5.8467	1.96E-08	1.33E-07	8.8279	CBS,CDO1,CSAD,CTH
N-Glycan Biosynthesis - Homo sapiens (human)	-0.5155	-6.9727	4.22E-11	2.28E-09	14.7837	AACS,ACAT1,ACAT2,BDH1,BDH2,HMGCL,HMGCS1,HMGCS2,OXCT1,OXCT2,HMGCS1,HMGCS2
Other Glycan Degradation - Homo sapiens (human)	-0.2530	-4.0150	8.35E-05	0.0002	0.8381	AADAT,AC-MSD,AFMID,GOT2,HAAO,IDO1,IDO2,KMO,KYAT1,KYAT3,KYNU,TDO2
Other Types of O-Glycan Biosynthesis - Homo sapiens (human)	-0.3161	-3.6299	0.0004	0.0007	-0.5266	AHCY,AMD1,BHMT,CARM1,CBS,CHDH,COMT,CTH,DNMT1,DNMT3A,DNMT3B,DOT1L,EZH2,GAMT,GNMT,HNMT,IL4I1,KMT5A,MARS,MAT1A,MAT2A,MAT2B,MRM2,MSRB2,MSRB3,MTAP,MTFMT,MTHFR,MTR,MTRR,NNMT,PCMT1,PEMT,PNMT,PRMT1,PRMT2,PRMT3,PRMT5,PRMT6,PRMT7,RNMT,SETD7,SETDB1,SHMT1,SMS,SMYD2,SRM,SUV39H1,SUV39H2
Arachidonic Acid Metabolism - Homo sapiens (human)	-0.0441	-0.7882	0.4315	0.4755	-6.5641	ALDOA,ALDOB,ALDOC,DERA,FBP1,FBP2,G6PD,GPI,H6PD,PFKL,PFKM,PFKP,PGD,PGLS,PGM1,PGM2,PRPS1,PRPS1L1,PRPS2,RBKS,RGN,RPE,RPIA,TALDO1,TKT,TKTL1,TKTL2,GLYCTK,IDNK,RPEL1
Biosynthesis of Unsaturated Fatty Acids - Homo sapiens (human)	-0.0154	-0.2751	0.7835	0.8136	-6.8349	GAPDH,GOT1,GOT2,GPD1,GPD2,MDH1,MDH2,NADK,NADK2,NNT,SLC1A3,SLC22A13,SLC25A11,SLC25A12,SLC25A13,SLC36A4,SLC5A8,SLC7A5
Ether Lipid Metabolism - Homo sapiens (human)	-0.2353	-4.0410	7.54E-05	0.0002	0.9344	BST1,CD38,APLF,CHFR,MACROD1,MACROD2,OARD1,PARG,PARP1,PARP10,PARP11,PARP12,PARP14,PARP15,PARP16,PARP2,PARP3,PARP4,PARP6,PARP8,PARP9,RNF146,TIPARP,TNKS,TNKS2,ZC3HAV1
Fatty Acid Biosynthesis - Homo sapiens (human)	-0.0964	-2.1292	0.0344	0.0454	-4.6436	NADSYN1,NMNAT1,NMNAT2,NMNAT3,QPRT,NAMPT,NAPRT,NMRK1,NMRK2
Fatty Acid Degradation - Homo sapiens (human)	-0.2166	-3.4174	0.0008	0.0013	-1.2279	QPRT,NAPRT,PNP,NMRK1,NMRK2,NT5C2,NT5C1A,NT5C1B,NT5C,NT5M,NT5C3A,NT5C3B,NT5C1B-RDH14,NT5E,NMNAT3,NMNAT1,NMNA

						T2,ENPP1,ENPP3,NUDT12,NADSYN1,NAMPT,CD38,BST1,NADK,NADK2,NNT,NNMT,AOX1
Fatty Acid Elongation - Homo sapiens (human)	-0.1801	-3.0181	0.0029	0.0046	-2.4431	SIRT1,SIRT2,SIRT3,SIRT4,SIRT5,SIRT6,SIRT7
Glycerolipid Metabolism - Homo sapiens (human)	0.1363	2.8764	0.0044	0.0067	-2.8411	AMD1,AZIN1,OAZ1,OAZ2,OAZ3,ODC1,PAOX,SAT1,SMOX,SMS,SRM,MAT2A,MAT2B
Glycerophospholipid Metabolism - Homo sapiens (human)	0.2573	2.9258	0.0038	0.0059	-2.7045	PTGS1,PTGS2,PTGIS
Linoleic Acid Metabolism - Homo sapiens (human)	0.1935	2.0655	0.0401	0.0522	-4.7736	TBXAS1
Primary Bile Acid Biosynthesis - Homo sapiens (human)	-0.2629	-5.1055	7.54E-07	2.71E-06	5.3160	ACACA,ACACB,ACAT1,ACAT2,ACOT12,ACSS1,ACSS2,ACYP1,ACYP2,AKR1B1,ALDH1B1,ALDH2,ALDH3A2,ALDH7A1,ALDH9A1,DLAT,DLD,GLO1,GRHPR,HAGH,HA-GHL,LDHA,LDHAL6A,LDHAL6B,LDHB,LDHC,LDHD,MDH1,MDH2,ME1,ME2,ME3,PC,PCK1,PCK2,PDHA1,PDHA2,PDHB,PDHX,PDK1,PDK2,PDK3,PDK4,PDP1,PDP2,PKLR,PKM,FH
Steroid Biosynthesis - Homo sapiens (human)	-0.2739	-3.8075	0.0002	0.0004	0.0881	ALDH1A1,ALDH1A2,ALDH1A3,CES1,CRABP1,CRABP2,CYP26A1,CYP26B1,CYP26C1,DHRS9,FABP5,LRAT,RBP1,RBP4,RDH10,RDH16,STRA6,TTR
Steroid Hormone Biosynthesis - Homo sapiens (human)	-0.1593	-2.4524	0.0150	0.0203	-3.9261	ABCA4,LRAT,OPN1LW,OPN1MW,OPN1SW,RBP1,RBP3,RDH10,RDH11,RDH12,RDH5,RDH8,RHO,RLBP1,RPE65
Biotin Metabolism - Homo sapiens (human)	-0.4137	-5.9346	1.25E-08	1.12E-07	9.2671	ABCA1,ADH1A,ADH1B,ADH1C,ADH4,ADH5,ADH6,ADH7,AKR1B10,ALDH1A1,ALDH1A2,AWAT1,AWAT2,BCO1,CD36,CYP1A1,CYP1A2,CYP26A1,CYP26B1,CYP26C1,CYP2A13,CYP2A6,CYP2A7,CYP2B6,CYP2C18,CYP2C19,CYP2C8,CYP2C9,CYP3A4,CYP3A43,CYP3A5,CYP3A7,CYP4A11,CYP4A22,DGAT1,DHRS3,DHRS4,DHRS4L2,DHRS9,DNAJB11,ERP29,HSP90B1,HSPA5,HYOU1,LRAT,PDIA2,PDIA4,PDIA6,PLB1,PNLIP,PNLIPRP2,PNPLA4,PPIB,RBP2,RDH10,RDH11,RDH12,RDH16,RDH5,RDH8,RETSAT,RPE65,SCARB1,SDF2L1,UGT1A1,UGT1A10,UGT1A3,UGT1A4,UGT1A5,UGT1A6,UGT1A7,AOX1,CYP2S1,CYP3A51P,DHRS4L1,HSD17B6,SDR16C5
Folate biosynthesis - Homo sapiens (human)	-0.3341	-5.3484	2.37E-07	9.84E-07	6.4275	AKR1C1,AKR1C2,AKR1C3,AKR1C4,AKR1D1,COMT,CYP11A1,CYP11B1,CYP11B2,CYP17A1,CYP19A1,CYP1A1,CYP1B1,CYP21A2,CYP3A4,CYP3A43,CYP3A5,CYP3A7,CYP7A1,CYP7B1,FDX1,FDXR,HSD11B1,HSD11B2,HSD17B1,HSD17B12,HSD17B2,HSD17B3,HSD17B6,HSD17B7,HSD17B8,HSD3B1,HSD3B2,POR,SRD5A1,SRD5A2,SRD5A3,STAR,STS,SULT1E1,SULT2B1,UGT1A1,UGT1A10,UGT1A3,UGT1A4,UGT1A5,UGT1A6,UGT1A7,UGT1A8,UGT1A9,UGT2A1,UGT2A2,UGT2A3,UGT2B10,UGT2B11,UGT2B15,UGT2B17,UGT2B28,UGT2B4,UGT2B7
Lipoic Acid Metabolism - Homo sapiens (human)	-0.1687	-3.0861	0.0023	0.0037	-2.2458	CYP11A1,CYP11B1,CYP11B2,CYP21A2,FDX1,FDXR,POR,STAR
Nicotinate and Nicotinamide Metabolism - Homo sapiens (human)	-0.3319	-4.9621	1.47E-06	4.53E-06	4.6781	CYP11B2,CYP11A1,CYP11B1,CYP17A1,CYP21A2,FDX1,FDXR,HSD3B1,HSD3B2,POR,STAR

Pantothenate and CoA Biosynthesis - Homo sapiens (human)	-0.3319	-4.9621	1.47E-06	4.53E-06	4.6781	CYP11A1,CYP17A1,CYP19A1,FDX1,FDXR,HSD17B1,HSD17B2,HSD17B3,HSD3B1,HSD3B2,POR,STAR,STS,SULT1E1,SULT2A1
Porphyrin and Chlorophyll Metabolism - Homo sapiens (human)	-0.3370	-5.4601	1.37E-07	6.45E-07	6.9517	CYP11A1,CYP17A1,FDX1,FDXR,HSD17B2,HSD17B3,HSD3B1,HSD3B2,POR,SRD5A1,SRD5A2,STAR,STS,SULT2A1
Retinol Metabolism - Homo sapiens (human)	-0.4235	-6.1269	4.54E-09	7.00E-08	10.2439	ARG1,ARG2,ASL,ASS1,CPS1,GLS2,GLUD1,GOT2,GPT,OTC,SLC1A4,SLC1A5,SLC25A12,SLC25A15
Riboflavin Metabolism - Homo sapiens (human)	-0.2584	-4.2223	3.64E-05	8.36E-05	1.6213	GGCX,VKORC1
Thiamine Metabolism - Homo sapiens (human)	-0.2693	-3.1704	0.0018	0.0029	-1.9956	KL,GUSB,UGT2A1,UGT2A3,UGT2B17,UGT2B11,UGT2B28,UGT1A6,UGT1A4,UGT1A1,UGT1A3,UGT2B10,UGT1A9,UGT2B7,UGT1A10,UGT1A8,UGT1A5,UGT2B15,UGT1A7,UGT2B4,UGT2A2,UGDH,UGP2,AKR1A1,CRYL1,RPE,RPEL1,XYLB,AKR1B1,AKR1B10,DCXR,SORD,DHDH,ALDH2,ALDH3A2,ALDH1B1
Ubiquinone and other Terpenoid-Quinone Biosynthesis - Homo sapiens (human)	-0.2376	-3.7711	0.0002	0.0004	-0.0399	MPI,PMM2,PMM1,GMPPB,GMPPA,GMDS,TSTA3,FPGT,FUK,ENOSF1,HK3,HK1,HK2,HKDC1,PFKM,PFKP,PFKL,FBP1,FBP2,PFKFB1,PFKFB2,PFKFB3,PFKFB4,TIGAR,KHK,SORD,AKR1B1,AKR1B10,ALDOC,ALDOA,ALDOB,TPI1,TKFC
Vitamin B6 Metabolism - Homo sapiens (human)	-0.0242	-0.4610	0.6453	0.6900	-6.7669	GALM,GALK1,GALT,GALE,UGP2,PGM1,PGM2,HK3,HK1,HK2,HKDC1,GCK,G6PC,G6PC2,G6PC3,GLB1,LCT,LALBA,B4GALT1,B4GALT2,GLA,AKR1B1,AKR1B10,PFKM,PFKP,PFKL,MGAM,MGAM2,GAA,GANC,SI
Beta-Alanine Metabolism - Homo sapiens (human)	0.0235	0.5610	0.5754	0.6277	-6.7162	UGDH,UGT2A1,UGT2A3,UGT2B17,UGT2B11,UGT2B28,UGT1A6,UGT1A4,UGT1A1,UGT1A3,UGT2B10,UGT1A9,UGT2B7,UGT1A10,UGT1A8,UGT1A5,UGT2B15,UGT1A7,UGT2B4,UGT2A2,MIOX,RGN,ALDH2,ALDH3A2,ALDH1B1,ALDH7A1,ALDH9A1
D-Glutamine and D-Glutamate Metabolism - Homo sapiens (human)	-0.3137	-5.5631	8.25E-08	4.69E-07	7.4425	MGAM,MGAM2,GAA,GANC,SI,UGP2,ENPP1,ENPP3,GBA3,GYS2,GYS1,GYG1,GYG2,GBE1,PYGL,PYGM,PYGB,AGL,AMY1C,AMY2A,AMY1A,AMY2B,AMY1B,TREH,PGM1,PGM2,HK3,HK1,HK2,HKDC1,GCK,G6PC,G6PC2,G6PC3,PGM2L1,GPI
Glutathione Metabolism - Homo sapiens (human)	-0.3434	-5.5478	8.90E-08	4.81E-07	7.3695	CHIA,CHIT1,HEXA,HEXB,NAGK,PGM3,UAP1,UAP1L1,GNE,RENBP,NANS,NANP,NPL,CMAS,CYB5R1,CYB5R3,CYB5R2,CYB5RL,CYB5R4,HK3,HK1,HK2,HKDC1,AMDHD2,GNPNAT1,GNPDA1,GNPDA2,GFPT2,GFPT1,UXS1,GCK,GPI,PGM1,PGM2,UGP2,UGDH,GALK1,GALT,GALE,PMM2,PMM1,GMPPB,GMPPA,GMDS,MPI,FUK,FPGT,TSTA3
Selenocompound Metabolism - Homo sapiens (human)	-0.1124	-1.2825	0.2011	0.2413	-6.0581	MDH1,MDH2,CS,ACO2,ACO1,ACAT2,ACAT1,MCEE,PCCA,PCCB,MUT,HAO2,HAO1,CAT,GRHPR,PGP,AGXT,GLUL,SHMT2,SHMT1,GLDC,AMT,DLD,GCSH,HYI,GLYCTK,HOGA1,AFMID
Taurine and Hypotaurine Metabolism - Homo sapiens (human)	-0.1154	-2.2826	0.0235	0.0313	-4.3151	ACSS1,ACSS2,ACSS3,BCKDHA,BCKDHB,DBT,DLD,ACADM,HADHA,EHHADH,ECHS1,HIBCH,ACACA,ACACB,MLYCD,ABAT,PCCA,PCCB,ECHDC1,MCEE,MUT,SUCLG1,SUCLG2,SUCLA2,ALDH6A1,LDHAL6A,LDHAL6B,LDHA,LDHB,LDHC,ACAT2,ACAT1

Terpenoid Backbone Biosynthesis - Homo sapiens (human)	-0.7586	-2.4568	0.0145	0.0567	-3.7915	ACAT2,ACAT1,HADH,HADHA,EHHADH,ECHS1,ACADS,ACSM1,ACSM2A,ACSM4,ACSM5,ACSM3,ACSM2B,ACSM6,L2HGDH,GAD1,GAD2,ABAT,ALDH5A1,HMGCS1,HMGCS2,HMGCL,HMGCLL1,OXCT1,OXCT2,AACS,BDH1,BDH2
Purine Metabolism - Homo sapiens (human)	-0.0660	-1.7179	0.0873	0.1084	-5.4159	PIK3C3,MTM1,MTMR1,MTMR2,MTMR3,MTMR4,MTMR8,MTMR6,MTMR7,MTMR14,PI4KA,PI4KB,PI4K2A,PI4K2B,PIP5K1C,PIP5K1A,PIP5K1B,PIP5KL1,OCRL,INPP5B,INPP5E,SYNJ1,SYNJ2,PIK3CA,PIK3CD,PIK3CB,PIK3CG,PTEN,INPP5D,INPPL1,PIK3C2G,PIK3C2A,PIK3C2B,INPP4A,INPP4B,PIKFYVE,PIP4K2C,PIP4K2A,PIP4K2B,PLCB1,PLCB2,PLCB3,PLCB4,PLCD1,PLCD3,PLCD4,PLCE1,PLCG1,PLCG2,PLCZ1,PLCH1,PLCH2,IMPA2,IMPA1,IMPAD1,CDIPT,ISYNA1,INPP1,INPP5K,INPP5J,INPP5A,MINPP1,ITPKB,IT-PKA,IT-PKC,ITPK1,IPMK,IPPK,MIOX,ALDH6A1,TPI1
Pyrimidine Metabolism - Homo sapiens (human)	-0.0645	-1.1722	0.2425	0.2786	-6.1917	ND1,ND2,ND3,ND4,ND4L,ND5,ND6,NDUFS1,NDUFS2,NDUFS3,NDUFS4,NDUFS5,NDUFS6,NDUFS7,NDUFS8,NDUFV1,NDUFV2,NDUFV3,NDUFA1,NDUFA2,NDUFA3,NDUFA4,NDUFA4L2,NDUFA5,NDUFA6,NDUFA7,NDUFA8,NDUFA9,NDUFA10,NDUFAB1,NDUFA11,NDUFA12,NDUFA13,NDUFB1,NDUFB2,NDUFB3,NDUFB4,NDUFB5,NDUFB6,NDUFB7,NDUFB8,NDUFB9,NDUFB10,NDUFB11,NDUFC1,NDUFC2,NDUFC2-KCTD14,SDHA,SDHB,SDHC,SDHD,UQCRFS1,CYTB,CYC1,UQCRC1,UQCRC2,UQCRH,UQCRHL,UQCRB,UQCRQ,UQCR10,UQCR11,COX10,COX3,COX1,COX2,COX4I2,COX4I1,COX5A,COX5B,COX6A1,COX11,COX15,COX17,ATP5A1,ATP5B,ATP5C1,ATP5D,ATP5E,ATP5O,ATP6,ATP5F1,ATP5G1,ATP5G2,ATP5G3,ATP5H,ATP5I,ATP5J2,ATP5L,ATP5J,ATP8,ATP6V1A,ATP6V1B1,ATP6V1B2,ATP6V1C2,ATP6V1C1,ATP6V1D,ATP6V1E2,ATP6V1E1,ATP6V1F,ATP6V1G1,ATP6V1G3,ATP6V1G2,ATP6V1H,TCIRG1,ATP6V0A2,ATP6V0A4,ATP6V0A1,ATP6V0C,ATP6V0B,ATP6V0D1,ATP6V0D2,ATP6V0E1,ATP6V0E2,ATP6AP1,ATP4A,ATP4B,ATP12A,PPA2,PPA1,LHPP
Metabolism of Xenobiotics by Cytochrome P450 - Homo sapiens (human)	-0.3568	-5.8774	1.68E-08	1.29E-07	8.9807	GLUD1,GLUD2,GLUL,CPS1,CA13,CA1,CA6,CA7,CA12,CA5B,CA14,CA9,CA3,CA5A,CA8,CA2,CA4
ADP-Ribosylation - Homo sapiens (human)	-0.2182	-3.5627	0.0005	0.0008	-0.7525	PAPSS1,PAPSS2,BPNT1,IM-PAD1,SUOX,CYCS,ETHE1,MPST,TST,SQRDL
Aldosterone Biosynthesis - Homo sapiens (human)	-0.3991	-5.8471	1.96E-08	1.33E-07	8.8299	FDFT1,SQLE,LSS,CYP51A1,TM7SF2,MSMO1,FAXDC2,NSDHL,HSD17B7,EBP,DHCR24,SC5D,DHCR7,LIPA,CEL,SOAT2,SOAT1,CYP2R1,CYP27B1,CYP24A1
alpha-Linoleic Acid Metabolism - Homo sapiens (human)	-0.4755	-6.4200	9.36E-10	3.37E-08	11.7736	CYP39A1,CYP46A,HSD3B7,CH25H,CYP7B1,CYP7A1,CYP27A1,CYP8B1,AKR1D1,AKR1C4,SLC27A5,AMACR,ACOX2,HSD17B4,SCP2,ACOT8,BAAT
Arginine and Proline Metabolism - Homo sapiens (human)	-0.2237	-3.7019	0.0003	0.0005	-0.2806	CYP17A1,CYP11A,STS,SULT2B1,CYP21A2,HSD3B1,HSD3B2,SRD5A1,SRD5A2,SRD5A3,AKR1C2,AKR1C3,CYP11B1,CYP11B2,AKR1D1,AKR1C4,HSD11B1,HSD11

						B2,AKR1C1,CYP7B1,SULT1E1,HSD17B1,HSD17B2,HSD17B6,HSD17B7,HSD17B8,HSD17B12,CYP1A1,CYP1A2,CYP3A5,CYP3A7,CYP3A7-CYP3A51P,CYP2E1,CYP3A4,CYP1B1,CYP19A1,CYP7A1,UGT2A1,UGT2A3,UGT2B17,UGT2B11,UGT2B28,UGT1A6,UGT1A4,UGT1A1,UGT1A3,UGT2B10,UGT1A9,UGT2B7,UGT1A10,UGT1A8,UGT1A5,UGT2B15,UGT1A7,UGT2B4,UGT2A2,COMT,HSD17B3
Ascorbate and Aldrate Metabolism - Homo sapiens (human)	-0.3653	-2.4341	0.0155	0.0596	-3.8456	GLYCTK,ALDH2,ALDH3A2,ALDH1B1,ALDH7A1,ALDH9A1,AKR1B1,AKR1B10,AKR1A1,TKFC,GK2,GK,GPAM,GPAT2,GPAT4,GPAT3,AGPAT1,AGPAT2,AGPAT5,LCLAT1,MBOAT1,MBOAT2,AGPAT3,AGPAT4,PLPP1,PLPP3,PLPP2,LPIN1,LPIN3,LPIN2,DGKZ,DGKD,DGKI,DGKA,DGKE,DGKB,DGKH,DGKG,DGKQ,DGKK,DGAT1,DGAT2,MOGAT3,LIPG,LIPC,CEL,PNPLA2,PNPLA3,PNLIP,PNLIPRP1,PNLIPRP2,PNLIPRP3,LIPF,LPL,AGK,MGLL,MOGAT1,MOGAT2,GLA
Caffeine Metabolism - Homo sapiens (human)	0.1486	3.2635	0.0013	0.0021	-1.7125	HPRT1,IMPDH1,IMPDH2,GMPS,TPMT,XDH,ITPA,CES1,CES2,UGT2A1,UGT2A3,UGT2B17,UGT2B11,UGT2B28,UGT1A6,UGT1A4,UGT1A1,UGT1A3,UGT2B10,UGT1A9,UGT2B7,UGT1A10,UGT1A8,UGT1A5,UGT2B15,UGT1A7,UGT2B4,UGT2A2,GUSB,CYP3A4,CDA,TYMP,DPYD,DPYS,UPB1,CYP2A6,UPP2,UPP1,UCK1,UCK2,UCKL1,TK2,TK1,UMPS,NAT2,NAT1
Cardiolipin Biosynthesis - Homo sapiens (human)	-0.5522	-2.4836	0.0135	0.0535	-3.7273	GPD1L,GPD1,GPD2,GPAM,GPAT2,GPAT4,GPAT3,AGPAT1,AGPAT2,AGPAT5,LCLAT1,MBOAT1,MBOAT2,AGPAT3,AGPAT4,GNPAT,ADPRM,PLPP1,PLPP3,PLPP2,LPIN1,LPIN3,LPIN2,DGKZ,DGKD,DGKI,DGKA,DGKE,DGKB,DGKH,DGKG,DGKQ,DGKK,CHPT1,CEPT1,PLD1,PLD2,PLD3,PLD4,LCAT,PLA2G10,PLA2G2D,PLA2G2E,PLA2G3,PLA2G2F,PLA2G12A,PLA2G12B,PLA2G1B,PLA2G5,PLA2G2A,PLA2G2C,PLA2G4E,PLA2G4A,JMJD7-PLA2G4B,PLA2G4B,PLA2G4C,PLA2G4D,PLA2G4F,PLA2G6,PLB1,PLA2G16,LPCAT2,LPCAT1,LPCAT4,LPCAT3,LY-PLA1,PLA2G15,LY-PLA2,PNPLA6,PNPLA7,GPCPD1,PEMT,CDS1,CDS2,PTDSS1,PTDSS2,PISD,PGS1,CRLS1,TAZ,LPGAT1,CDIPT,MBOAT7
Cardiolipin Metabolism - Homo sapiens (human)	0.0055	0.1503	0.8807	0.8973	-6.8613	PLPP1,AGPS,PLPP3,PLPP2,EPT1,CHPT1,CEPT1,PLA2G10,PLA2G2D,PLA2G2E,PLA2G3,PLA2G2F,PLA2G12A,PLA2G12B,PLA2G1B,PLA2G5,PLA2G2A,PLA2G2C,PLA2G4E,PLA2G4A,JMJD7-PLA2G4B,PLA2G4B,PLA2G4C,PLA2G4D,PLA2G4F,PLA2G6,PLB1,PLA2G16,LPCAT4,ENPP6,ENPP2,TMEM86B,PLD1,PLD2,PLD3,PLD4,UGT8,GAL3ST1,LPCAT2,LPCAT1,PAFAH1B1,PAFAH1B2,PAFAH1B3,PLA2G7,PAFAH2
Cholesterol Biosynthesis - Homo sapiens (human)	0.0732	1.8823	0.0612	0.0787	-5.1263	SPTLC1,SPTLC2,SPTLC3,KDSR,CERS2,CERS3,CERS6,CERS1,CERS4,CERS5,ASAH1,ASAH2,ACER2,ACER1,ACER3,DEGS1,DEGS2,SGMS1,SGMS2,SMPD1,SMPD2,SMPD3,SMPD4,ENPP7,CERK,PLPP1,PLPP3,PLPP2,SGPP1,SGPP2,SPHK1,SPHK2,SGPL1,UGCG,GBA,GBA2,B4GALT6,G

						LB1,UGT8,GALC,GAL3ST1,ARSA,NEU1,NEU3,NEU4,NEU2,GLA
Citric Acid Cycle - Homo sapiens (human)	-0.0185	-0.3922	0.6953	0.7362	-6.7961	PLA2G10,PLA2G2D,PLA2G2E,PLA2G3,PLA2G2F,PLA2G12A,PLA2G12B,PLA2G1B,PLA2G5,PLA2G2A,PLA2G2C,PLA2G4E,PLA2G4A,JMJD7-PLA2G4B,PLA2G4B,PLA2G4C,PLA2G4D,PLA2G4F,PLA2G6,PLB1,PLA2G16,PTGS1,PTGS2,PTGES,PTGES2,PTGES3,CBR1,CBR3,FAM213B,TBXAS1,PTGDS,HPGDS,AKR1C3,PTGIS,ALOX5,LTA4H,CYP4F2,CYP4F3,LTC4S,GGT1,GGT5,GPX6,GPX7,GPX2,GPX3,GPX1,GPX5,GPX8,CYP2E1,CYP2J2,CYP2U1,CYP4A11,CYP2C19,CYP4F8,ALOX12,ALOX12B,ALOX15B,CYP2B6,CYP2C8,CYP2C9,EPHX2,ALOX15
Cyclooxygenase Arachidonic Acid Metabolism - Homo sapiens (human)	-0.1302	-2.8847	0.0043	0.0066	-2.8183	PLA2G10,PLA2G2D,PLA2G2E,PLA2G3,PLA2G2F,PLA2G12A,PLA2G12B,PLA2G1B,PLA2G5,PLA2G2A,PLA2G2C,PLA2G4E,PLA2G4A,JMJD7-PLA2G4B,PLA2G4B,PLA2G4C,PLA2G4D,PLA2G4F,PLA2G6,PLB1,PLA2G16,ALOX15,CYP1A2,CYP2C8,CYP2C9,CYP2C19,CYP2J2,CYP2E1,CYP3A4
Dopamine Biosynthesis - Homo sapiens (human)	-0.4238	-2.4587	0.0145	0.0565	3.5953	ELOVL6,HSD17B12,HACD2,HACD1,HACD4,HACD3,TECR,PECR,SCD,SCD5,FADS2,ELOVL5,FADS1,ELOVL2,ACOX3,ACOX1,HADHA,ACAA1,ACOT4,ACOT2,ACOT1,ACOT7,BAAT
Drug Metabolism by Cytochrome P450 - Homo sapiens (human)	-0.2298	-2.6275	0.0093	0.0135	-3.4976	NUDT9,ADPRM,NUDT5,PGM1,PGM2,PRPS1L1,PRPS2,PRPS1,PPAT,GART,PFAS,PAICS,ADSL,ATIC,APRT,NT5C2,NT5C1A,NT5C1B,NT5C,NT5M,NT5C3A,NT5C3B,NT5C1B-RDH14,NT5E,PNP,HPRT1,IMPDH1,IMPDH2,NME6,NME7,NME2,NME4,NME1,NME3,NME1-NME2,AK9,ENTPD3,ENTPD8,ENTPD1,CANT1,ENTPD4,ENTPD5,ENTPD6,NUDT16,ITPA,XDH,NUDT2,GMPS,GMPR,GMPR2,GDA,GUK1,PKM,PKLR,RRM1,RRM2B,RRM2,DGUOK,POLR1A,POLR1B,ZNRD1,TWISTNB,POLR1E,POLR2A,POLR2B,POLR2C,POLR2D,POLR2E,POLR2F,POLR2G,POLR2H,POLR1C,POLR3K,POLR1D,POLR3H,POLR3GL,POLR3G,POLR3F,POLA1,POLA2,PRIM1,PRIM2,POLD1,POLD2,POLD3,POLD4,POLE,POLE2,POLE3,POLE4,HDDC3,PRUNE1,ADCY1,ADCY2,ADCY3,ADCY4,ADCY5,ADCY6,ADCY7,ADCY8,ADCY9,ADCY10,GUCY1A2,GUCY1A3,GUCY1B3,GUCY2C,GUCY2D,GUCY2F,NPR1,NPR2,PDE1A,PDE1B,PDE1C,PDE2A,PDE3A,PDE3B,PDE5A,PDE6A,PDE6B,PDE6C,PDE6D,PDE6G,PDE6H,PDE9A,PDE10A,PDE11A,AD-SSL1,ADSS,AMPD2,AMPD3,AMPD1,ADK,DCK,ADA,CECR1,AK7,AK4,AK5,AK2,AK1,adenyl-ate,AK8,AK6,AK3,ENTPD2,NTPCR,PNPT1,PDE4A,PDE4B,PDE4C,PDE4D,PDE7A,PDE7B,PDE8B,PDE8A,FHIT,ENPP4,PAPSS2,PAPSS1,ENPP1,ENPP3,URAD,ALLCAD,DHODH,UMPS,CMPK1,CMPK2,NME6,NME7,NME2,NME4,NME1,NME3,NME1-
Drug Metabolism by other enzymes - Homo sapiens (human)	-0.1559	-2.5624	0.0111	0.0158	-3.6602	NME2,AK9,PNPT1,ENTPD3,ENTPD8,ENTPD1,CANT1,ENTPD4,ENTPD5,ENTPD6

						,CTPS1,CTPS2,POLR1A,POLR1B,ZNRD1,TWISTNB,POLR1E,POLR2A,POLR2B,POLR2C,POLR2D,POLR2E,POLR2F,POLR2G,POLR2H,POLR2I,POLR2L,POLR2J,POLR2J3,POLR2J2,POLR2K,POLR3A,POLR3B,POLR3C,POLR3D,POLR3E,RPC5,POLR1C,POLR3K,POLR1D,POLR3H,POLR3GL,POLR3G,POLR3F,POLA1,POLA2,PRIM1,PRIM2,POLD1,POLD2,POLD3,POLD4,POLE,POLE2,POLE3,POLE4,UCK1,UCK2,UCKL1,NT5C2,DPYS,UPB1,ENPP1,ENPP3,TXNRD1,TXNRD2,TXNRD3,RRM1,RRM2B,RRM2,DCTPP1,DUT,TYMS,CDA,TYMP,PNP,DCK,DCTD,TK2,TK1,DTYMK,NUDT2
Epinephrine Biosynthesis - Homo sapiens (human)	-0.2563	-5.1675	5.63E-07	2.10E-06	5.5958	GOT1,GOT2,IL4I1,DDO,ASNS,NIT2,GPT2,GPT,AGXT,AGXT2,ASS1,ASL,ADSSL1,ADSS,ADSL,NAT8L,RIMKLB,RIMKLA,FOLH1,ASPA,GAD1,GAD2,ABAT,ALDH5A1,GLUD2,GLUD1,ALDH4A1,GLUL,CAD,GLS2,GLS,CPS1,GFPT2,GFPT1,PPAT
Phenylalanine Metabolism - Homo sapiens (human)	-0.4525	-6.0418	7.11E-09	9.60E-08	9.8091	ADSS,ADSSL1,AK1,AK2,AK3,AK4,AK5,ATIC,GART,GMPS,IMPDH1,IMPDH2,PAICS,PFAS,PPAT,PRPS1,PRPS1L1,PRPS2,TAF9
Folate One Carbon Metabolism - Homo sapiens (human)	0.0481	0.9568	0.3398	0.3823	-6.4185	CTH,CBS,CBSL,BHMT,BHMT2,MTR,MAT2B,MAT1A,MAT2A,AMD1,SRM,SMS,MTAP,MRI1,APIP,ENOPH1,ADI1,TAT,IL4I1,DNMT1,DNMT3A,DNMT3B,AHCYL2,AHCYL1,AHCY,BCAT2,BCAT1,AGXT2,GCLC,GCLM,GSS,CDO1,GOT1,GOT2,MPST,TST,LDHAL6A,LDHAL6B,LDHA,LDHB,LDHC,MDH1,MDH2,SDS,SDSL
Gluconeogenesis - Homo sapiens (human)	-0.2792	-4.6995	4.79E-06	1.29E-05	3.5475	BCAT2,BCAT1,IL4I1,BCKDHA,BCKDHB,DBT,DLD,ACADS,ACADM,IVD,ACADSB,ACAD8,HADHA,EHHADH,ECHS1,HADH,HSD17B10,ACAA1,ACAA2,HADHB,PCCA,PCCB,MCEE,MUT,HIBCH,HIBADH,ALDH6A1,ALDH2,ALDH3A2,ALDH1B1,ALDH7A1,ALDH9A1,AOX1,ACSF3,ABAT,AGXT2,MCCC1,MCCC2,AUH,HMGCL,HMGCLL1,OXCT1,OXCT2,AACS,ACAT2,ACAT1,HMGCS1,HMGCS2
Glycogen Biosynthesis - Homo sapiens (human)	0.0198	0.5208	0.6031	0.6513	-6.7378	SDS,SDSL,BCAT2,BCAT1
Glycogen Degradation - Homo sapiens (human)	1.0175	2.4349	0.0154	0.0595	-3.8437	AASS,ALDH7A1,AADAT,OGDHL,OGDH,DLST,GCDH,HADHA,EHHADH,ECHS1,HADH,ACAT2,ACAT1,HYKK,PHYKPL,PIPOX,ASH1L,DOT1L,EHMT2,EHMT1,KMT2A,KMT2D,KMT2C,KMT2B,KMT2E,NSD1,WHSC1,WHSC1L1,SETD1B,SETD1A,SETD2,SETD3,SETD7,KMT5A,SETDB1,SETDB2,SETMAR,SUV39H1,SUV39H2,KMT5C,KMT5B,EZH1,EZH2,PRDM2,PRDM6,PRDM9,PRDM7,CAMKMT,TMLHE,ALDH2,ALDH3A2,ALDH1B1,ALDH9A1,BBOX1,PLOD1,PLOD2,PLOD3,COLGALT1,COLGALT2
Glycolysis - Homo sapiens (human)	-0.4143	-5.5185	1.03E-07	5.05E-07	7.2295	OTC,ASS1,ASL,ARG2,ARG1,NOS1,NOS2,NOS3,GLS2,GLS,GLUL,GLUD2,GLUD1,CPS1,GOT1,GOT2,GPT2,GPT,NAGS,ACY1,ABHD14A-ACY1
Glycosaminoglycan Biosynthesis - Homo sapiens (human)	-0.0694	-1.8590	0.0645	0.0819	-5.1690	GAMT,GATM,CKM,CKMT1A,CKMT2,CKB,CKMT1B,AZIN2,AGMAT,ODC1,SRM,SMS,AMD1,AOC1,SMOX,ALDH2,ALDH3A2,ALDH1B1,ALDH7A1,ALDH9A1,CNDP1,CNDP2,CARNS1,S

						AT2,SAT1,MAOB,MAOA,NOS1,NOS2,NOS3,ARG2,ARG1,OAT,PYCRL,PYCR2,PYCR1,PRODH,ALDH4A1,ALDH18A1,LAP3,P4HA2,P4HA3,P4HA1,PRODH2,GOT1,GOT2,HOGA1,DAO,L3HYPDH
Glycosphingolipid Biosynthesis - Homo sapiens (human)	-0.1934	-4.1183	5.54E-05	0.0001	1.2240	HAL,UROC1,AMDHD1,FTCD,HDC,DDC,AOC1,ALDH2,ALDH3A2,ALDH1B1,ALDH7A1,ALDH9A1,ASPA,HNMT,MAOB,MAOA,ALDH3B1,ALDH3B2,ALDH1A3,ALDH3A1,CARNS1,CARNMT1,CNDP2,CNDP1
Glycosphosphatidylinositol - Homo sapiens (human)	-0.2423	-5.2084	4.64E-07	1.79E-06	5.7822	GOT1,GOT2,TAT,IL4I1,HPD,HGD,GSTZ1,FAH,TYR,TH,DCT,TYRP1,DDC,DBH,PNMT,COMT,MAOB,MAOA,AOC3,AOC2,ALDH3B1,ALDH3B2,ALDH1A3,ALDH3A1,ADH1A,ADH1B,ADH1C,ADH7,ADH4,ADH5,ADH6,TPO,AOX1,FAHD1,MIF
Heme Biosynthesis - Homo sapiens (human)	-0.3312	-5.4438	1.49E-07	6.69E-07	6.8750	PAH,DDC,AOC3,AOC2,MAOB,MAOA,ALDH3B1,ALDH3B2,ALDH1A3,ALDH3A1,GLYAT,GOT1,GOT2,TAT,IL4I1,HPD,MIF
Hexosamine Biosynthesis - Homo sapiens (human)	-0.2988	-5.0606	9.30E-07	3.24E-06	5.1145	IDO1,TDO2,IDO2,AFMID,KMO,KYNU,HAAO,ACMSD,OGDHL,OGDH,GCDH,HADHA,EHHADH,ECHS1,HADH,ACAT2,ACAT1,KYAT3,KYAT1,AADAT,TPH2,TPH1,DDC,MAOB,MAOA,ALDH2,ALDH3A2,ALDH1B1,ALDH7A1,ALDH9A1,AOX1,ASMT,AANAT,CYP1A1,CYP1A2,CYP1B1,INMT,IL4I1,AOC1,CAT
Homocysteine Biosynthesis - Homo sapiens (human)	-0.3959	-6.1402	4.23E-09	7.00E-08	10.3124	GOT1,GOT2,TAT,IL4I1,PAH
Ketone Biosynthesis and Metabolism - Homo sapiens (human)	-0.3540	-6.1402	4.23E-09	7.00E-08	10.3123	GADL1,GAD1,GAD2,CNDP1,CARNS1,CNDP2,ABAT,SRM,SMS,SMOX,AOC3,AOC2,ALDH2,ALDH3A2,ALDH1B1,ALDH7A1,ALDH9A1,ALDH3B1,ALDH3B2,ALDH1A3,ALDH3A1,DPYD,DPYS,UPB1,HIBCH,HADHA,EHHADH,ECHS1,ACADM,MLYCD,ALDH6A1
Kynurenine Metabolism - Homo sapiens (human)	-0.0598	-1.2267	0.2213	0.2598	-6.1271	CDO1,GAD1,GAD2,CSAD,GADL1,ADO,GGT7,GGT6,GGT1,GGT5,BAAT
Methionine Cycle - Homo sapiens (human)	-0.1264	-2.4995	0.0132	0.0181	-3.8135	MTR,CTH,SCLY,KYAT3,KYAT1,TXNRD1,TXNRD2,TXNRD3,INMT,PAPSS2,PAPSS1,SEPHS2,SEPHS1,PSTK,SEPSECS,MARS,MARS2
Neomycin, Kanamysin and Gentamicin Biosynthesis - Homo sapiens (human)	-0.4543	-2.4384	0.0153	0.0590	-3.8353	GLS2,GLUD2,GLUD1
Nicotinamide Adenine Dinucleotide Biosynthesis - Homo sapiens (human)	-7.7604	-2.4356	0.0154	0.0594	-3.8420	GGT7,GGT6,GGT1,GGT5,GGCT,OPLAH,GCLC,GCLM,GSS,LAP3,ANPEP,GSTA5,GSTA2,GSTA4,GSTO2,GSTM4,GSTT2,GSTT1,GSTM3,MGST1,MGST3,GSTP1,GSTM1,GSTM5,MGST2,GSTA1,GSTM2,GSTA3,GSTO1,GSTT2B,GSTK1,HPGDS,NAT8,NAT8B,GSR,IDH1,IDH2,PGD,G6PD,TXNDC12,GPX6,GPX7,GPX2,GPX3,GPX1,GPX5,GPX8,GPX4,ODC1,SRM,SMS,RRM1,RRM2B,RRM2
Nicotinamide Adenine Metabolism - Homo sapiens (human)	-0.0041	-0.1117	0.9112	0.9197	-6.8664	DOLK,DPAGT1,ALG5,ALG13,ALG14,DPM1,DPM2,DPM3,ALG1,ALG2,ALG11,ALG3,ALG9,ALG12,ALG6,ALG8,ALG10,ALG10B,STT3A,STT3B,RPN1,RPN2,DAD1,TUSC3,DDOST,DOLPP1,MOGS,GANAB,MAN1B1,MAN1A2,MAN1C1,MAN1A1,MGAT1,MAN2A1,MAN2A2,MGAT2,FUT8,B4GALT1,B4GALT2,B4GALT3,ST

						6GAL1,ST6GAL2,MGAT3,MGAT4A,MGAT4B,MGAT4D,MGAT5,MGAT5B,MGAT4C
Norepinephrine Biosynthesis - Homo sapiens (human)	-0.0749	-1.5527	0.1220	0.1481	-5.6810	GALNT5,GALNTL6,WBSCR17,GALNT11,GALNT12,GALNT13,GALNT14,GALNT16,GALNT15,GALNT18,GALNTL5,GALNT10,GALNT2,GALNT3,GALNT1,GALNT6,GALNT4,GALNT9,GALNT7,GALNT8,POC1B-GALNT4,C1GALT1,C1GALT1C1,GCNT1,GCNT3,GCNT4,ST3GAL1,ST3GAL2,ST6GALNAC1,B3GNT6,B4GALT5
Oxidative Phosphorylation - Homo sapiens (human)	0.9991	2.4773	0.0137	0.0542	3.7569	POMT1,POMT2,POMGNT1,B4GALT1,B4GALT2,B4GALT3,ST3GAL3,FUT9,FUT4,FUT7,MGAT5B,B3GALT4,B3GAT1,B3GAT2,CHST10,OGT,EOGT,POFUT1,POFUT2,MFNG,LFNG,RFNG,ST6GAL1,ST6GAL2,B3GLCT,POGLUT1,GXYLT1,GXYLT2,COLGALT1,COLGALT2,PLOD3
Pentose Phosphate - Homo sapiens (human)	-0.1366	-2.5476	0.0116	0.0162	-3.6966	XYLT2,XYLT1,B4GALT7,B3GALT6,B3GAT3,CSGALNACT1,CSGALNACT2,CHSY3,CHSY1,CHPF,CHPF2,DSE,CHST11,CHST12,CHST13,CHST3,CHST7,CHST15,UST,CHST14,EXTL2,EXTL3,EXTL1,EXT1,EXT2,NDST1,NDST2,NDST3,NDST4,GLCE,HS2ST1,HS6ST1,HS6ST2,HS6ST3,HS3ST1,HS3ST2,HS3ST3B1,HS3ST3A1,HS3ST5,B4GALT1,FUT8,B4GALT2,B4GALT3,B3GNT2,B4GAT1,CHST6,B4GALT4,B3GNT7,CHST1,ST3GAL3,CHST2,CHST4,ST3GAL1,ST3GAL2
Polyamine Biosynthesis - Homo sapiens (human)	-0.4445	-5.4355	1.55E-07	6.69E-07	6.8356	HYAL2,HYAL1,SPAM1,HYAL4,HYAL3,GUSB,IDS,IDUA,ARSB,HPSE,HPSE2,SGSH,HGSNAT,NAGLU,GALNS,GLB1,GNS,HEXA,HEXB
Prostaglandin Biosynthesis - Homo sapiens (human)	0.0513	1.2593	0.2094	0.2485	-6.0872	PIGA,PIGH,PIGC,PIGQ,PIGP,PIGY,DPM2,PIGL,PIGW,GPLD1,PIGM,PIGX,PIGV,PIGN,PIGB,PIGF,PIGO,GPAA1,PIGK,PIGS,PIGT,PIGU,PGAP1,PIGZ,PIGG
Prostanoid Biosynthesis - Homo sapiens (human)	-3.6314	-2.5091	0.0126	0.0506	-3.6656	B3GALT1,B3GNT5,B3GALT2,B3GALT5,FUT1,FUT2,FUT3,ST3GAL3,ST3GAL4,ABO,B4GALT1,B4GALT2,B4GALT3,B4GALT4,FUT9,FUT4,FUT5,FUT6,FUT7,ST3GAL6,ST8SIA1,B3GNT2,B4GAT1,B3GNT3,B3GNT4,GCNT2,A3GALT2,B3GALNT1,GBGT1,A4GALT,GLA,HEXA,HEXB,NAGA,ST3GAL1,ST3GAL2,B4GALNT1,B3GALT4,ST8SIA5,ST3GAL5,SLC33A1,ST6GALNAC3,ST6GALNAC4,ST6GALNAC5,ST6GALNAC6,GLB1
Purine Biosynthesis - Homo sapiens (human)	-2.8639	-2.4325	0.0155	0.0598	-3.8493	NEU1,NEU3,NEU4,NEU2,GLB1,HEXA,HEXB,HEXDC,MAN2C1,MAN2B1,MAN2B2,MANBA,ENGASE,FUCA1,FUCA2,AGA,GBA,GBA2
Pyrimidine Biosynthesis - Homo sapiens (human)	-1.8139	-2.4328	0.0155	0.0597	-3.8485	NFS1,NTPCR,TPK1,THTPA
Remethylation - Homo sapiens (human)	38.8873	2.4336	0.0155	0.0597	-3.8467	RFK,FLAD1,BLVRB,TYR
Retinoic Acid Metabolism - Homo sapiens (human)	-0.6576	-2.4945	0.0131	0.0522	-3.7011	PNPO,PDXK,PDXP,PHOSPHO2,AOX1,PSAT1
Retinoid Metabolism - Homo sapiens (human)	-0.0416	-0.8597	0.3910	0.4353	-6.5058	PANK4,PANK1,PANK3,PANK2,PPCS,PPCDC,ENPP1,ENPP3,COASY,AASDHPPT,VNN1,VNN2,BCAT2,BCAT1,DPYD,DPYS,UPB1,GADL1

Shingolipid Metabolism - Homo sapiens (human)	-0.2829	-3.7776	0.0002	0.0004	-0.0174	OXSM,HLCS,BTD
Sirtuin Nicotinamide Metabolism - Homo sapiens (human)	0.0497	1.1036	0.2711	0.3082	-6.2688	LIAS,LIPT2,LIPT1
Starch and Suctose Metabolism - Homo sapiens (human)	-0.4873	-2.4327	0.0155	0.0597	-3.8488	ALAS2,ALAS1,EARS2,EPRS,ALAD,HMBS,UROS,UROD,CPOX,PPOX,FECH,COX10,COX15,MMAB,HMOX1,HMOX2,BLVRA,BLVRB,UGT2A1,UGT2A3,UGT2B17,UGT2B11,UGT2B28,UGT1A6,UGT1A4,UGT1A1,UGT1A3,UGT2B10,UGT1A9,UGT2B7,UGT1A10,UGT1A8,UGT1A5,UGT2B15,UGT1A7,UGT2B4,UGT2A2,GUSB,HCCS,CP,HEPH,FXN
Steroid Hormone Metabolism - Homo sapiens (human)	-0.2676	-4.9035	1.92E-06	5.75E-06	4.4218	TAT,COQ2,COQ3,COQ6,COQ5,COQ7,NQO1,GGCX,VKORC1,VKORC1L1,HPD
Thromboxane Biosynthesis - Homo sapiens (human)	-0.4084	-4.6590	5.72E-06	1.51E-05	3.3774	CYP1A2,NAT2,NAT1,CYP2A6,XDH
Transsulfuration - Homo sapiens (human)	9.4176	2.4333	0.0155	0.0597	-3.8474	GALM,GALK1,GALT,GALE,UGP2,PGM1,PGM2,HK3,HK1,HK2,HKDC1,GCK,G6PC,G6PC2,G6PC3,GLB1,LCT,LALBA,B4GALT1,B4GALT2,GLA,AKR1B1,AKR1B10,PFKM,PFKP,PFKL,MGAM,MGAM2,GAA,GANC,SI
Urea Cycle - Homo sapiens (human)	-0.2847	-4.2077	3.86E-05	8.69E-05	1.5650	CYP1A1,CYP2C9,CYP3A4,CYP1B1,GSTA5,GSTA2,GSTA4,GSTO2,GSTM4,GSTT2,GSTT1,GSTM3,MGST1,MGST3,GSTP1,GSTM1,GSTM5,MGST2,GSTA1,GSTM2,GSTA3,GSTO1,GSTT2B,GSTK1,HPGDS,EPHX1,CYP2B6,SULT2A1,CYP1A2,CYP2A6,CYP2E1,CYP2F1,CYP2S1,AKR1C1,DHDH,CYP2A13,CYP2D6,HSD11B1,CBR1,CBR3,UGT2A1,UGT2A3,UGT2B17,UGT2B11,UGT2B28,UGT1A6,UGT1A4,UGT1A1,UGT1A3,UGT2B10,UGT1A9,UGT2B7,UGT1A10,UGT1A8,UGT1A5,UGT2B15,UGT1A7,UGT2B4,UGT2A2,CYP3A5,AKR7A2,AKR7A3,ALDH3B1,ALDH3B2,ALDH1A3,ALDH3A1,ADH1A,ADH1B,ADH1C,ADH7,ADH4
Vitamin K - Homo sapiens (human)	0.0947	1.1759	0.2410	0.2786	-6.1874	CYP2C9,CYP2D6,CYP3A4,FMO1,FMO2,FMO5,FMO3,FMO4,CYP2C19,CYP2B6,CYP3A5,GSTA5,GSTA2,GSTA4,GSTO2,GSTM4,GSTT2,GSTT1,GSTM3,MGST1,MGST3,GSTP1,GSTM1,GSTM5,MGST2,GSTA1,GSTM2,GSTA3,GSTO1,GSTT2B,GSTK1,HPGDS,ADH1A,ADH1B,ADH1C,ADH7,ADH4,ADH5,ADH6,ALDH3B1,ALDH3B2,ALDH1A3,ALDH3A1,MAOB,MAOA,AOX1,UGT2A1,UGT2A3,UGT2B17,UGT2B11,UGT2B28,UGT1A6,UGT1A4,UGT1A1,UGT1A3,UGT2B10,UGT1A9,UGT2B7,UGT1A10,UGT1A8,UGT1A5,UGT2B15,UGT1A7,UGT2B4,UGT2A2,CYP1A2,CYP2E1,CYP2C8,CYP2A6

Note: [1] from Rosario, S. R., et al. (doi: 10.1038/s41467-018-07232-8).

Table S5. DEGs located in the copy number variation regions .

	Gene Name	Gene Symbol	Entrez Gene ID	regulated	Chromosome
**amplified regions**	oxidative stress induced growth inhibitor family member 2	OSGIN2	734	up	8q21.13
	MYB proto-oncogene like 1	MYBL1	4603	up	8q21.13
	nibrin	NBN	4683	up	8q21.13
	ribosomal protein L7	RPL7	6129	up	8q21.13
	ribosomal protein L30	RPL30	6156	up	8q21.13
	serine/threonine kinase 3	STK3	6788	up	8q21.13
	tumor protein D52	TPD52	7163	up	8q21.13
	ubiquinol-cytochrome c reductase binding protein	UQCRB	7381	up	8q21.13
	receptor interacting serine/threonine kinase 2	RIPK2	8767	up	8q21.13
	cyclin E2	CCNE2	9134	up	8q21.13
	phosphatidylserine synthase 1	PTDSS1	9791	up	8q21.13
	POP1 homolog, ribonuclease P/MRP subunit	POP1	10940	up	8q21.13
	ribosome biogenesis regulator homolog	RRS1	23212	up	8q21.13
	lymphocyte antigen 96	LY96	23643	up	8q21.13
	RAD54 homolog B	RAD54B	25788	up	8q21.13
	ganglioside induced differentiation associated protein 1	GDAP1	54332	up	8q21.13
	pyruvate dehyrogenase phosphatase catalytic subunit 1	PDP1	54704	up	8q21.13
	lysosomal protein transmembrane 4 beta	LAPTM4B	55353	up	8q21.13
	integrator complex subunit 8	INTS8	55656	up	8q21.13
	sorting nexin 16	SNX16	64089	up	8q21.13
	centrosome and spindle pole associated protein 1	CSPP1	79848	up	8q21.13
	phosphatidylinositol-3,4,5-trisphosphate dependent Rac exchange factor 2	PREX2	80243	down	8q21.13
	alcohol dehydrogenase, iron containing 1	ADHFE1	137872	down	8q21.13
	RNA binding motif protein 12B	RBM12B	389677	up	8q21.13
	Pvt1 oncogene	PVT1	5820	up	8q24.13
	ATPase family, AAA domain containing 2	ATAD2	29028	up	8q24.13
	tRNA methyltransferase 12 homolog	TRMT12	55039	up	8q24.13
	WDYHV motif containing 1	WDYHV1	55093	up	8q24.13
	NSE2/MMS21 homolog, SMC5-SMC6 complex SUMO ligase	NSMCE2	286053	up	8q24.13
**deleted regions**	RB transcriptional corepressor 1	RB1	5925	down	13q14.2
	ceroid-lipofuscinosis, neuronal 5	CLN5	1203	down	13q22.2
	endothelin receptor type B	EDNRB	1910	down	13q22.2
	LIM domain 7	LMO7	4008	down	13q22.2
	progesterone immunomodulatory binding factor 1	PIBF1	10464	down	13q22.2
	Kruppel like factor 12	KLF12	11278	down	13q22.2
	DIS3 homolog, exosome endoribonuclease and 3'-5' exoribonuclease	DIS3	22894	down	13q22.2
	MYC binding protein 2, E3 ubiquitin protein ligase	MYCBP2	23077	down	13q22.2
	tudor domain containing 3	TDRD3	81550	down	13q22.2
	mitogen-activated protein kinase kinase 3	MAP2K3	5606	down	17p11.2
	dehydrogenase/reductase 7B	DHRS7B	25979	down	17p11.2
